# Exploring dynamic metabolomics data with multiway data analysis: a simulation study

**DOI:** 10.1186/s12859-021-04550-5

**Published:** 2022-01-10

**Authors:** Lu Li, Huub Hoefsloot, Albert A. de Graaf, Evrim Acar, Age K. Smilde

**Affiliations:** 1 Machine Intelligence Department, Simula Metropolitan Center for Digital Engineering, Oslo, Norway; 2 Swammerdam Institute for Life Sciences, University of Amsterdam, Amsterdam, The Netherlands; 3 Netherlands Organisation for Applied Scientific Research (TNO), Zeist, The Netherlands

**Keywords:** Dynamic metabolomics data, Tensor factorization, CANDECOMP/PARAFAC, Paralind

## Abstract

**Background:**

Analysis of dynamic metabolomics data holds the promise to improve our understanding of underlying mechanisms in metabolism. For example, it may detect changes in metabolism due to the onset of a disease. Dynamic or time-resolved metabolomics data can be arranged as a three-way array with entries organized according to a *subjects* mode, a *metabolites* mode and a *time* mode. While such time-evolving multiway data sets are increasingly collected, revealing the underlying mechanisms and their dynamics from such data remains challenging. For such data, one of the complexities is the presence of a superposition of several sources of variation: induced variation (due to experimental conditions or inborn errors), individual variation, and measurement error. Multiway data analysis (also known as tensor factorizations) has been successfully used in data mining to find the underlying patterns in multiway data. To explore the performance of multiway data analysis methods in terms of revealing the underlying mechanisms in dynamic metabolomics data, simulated data with known ground truth can be studied.

**Results:**

We focus on simulated data arising from different dynamic models of increasing complexity, i.e., a simple linear system, a yeast glycolysis model, and a human cholesterol model. We generate data with induced variation as well as individual variation. Systematic experiments are performed to demonstrate the advantages and limitations of multiway data analysis in analyzing such dynamic metabolomics data and their capacity to disentangle the different sources of variations. We choose to use simulations since we want to understand the capability of multiway data analysis methods which is facilitated by knowing the ground truth.

**Conclusion:**

Our numerical experiments demonstrate that despite the increasing complexity of the studied dynamic metabolic models, tensor factorization methods CANDECOMP/PARAFAC(CP) and Parallel Profiles with Linear Dependences (Paralind) can disentangle the sources of variations and thereby reveal the underlying mechanisms and their dynamics.

**Supplementary Information:**

The online version contains supplementary material available at 10.1186/s12859-021-04550-5.

## Background

With the availability of advanced analytical measurement techniques such as Nuclear Magnetic Resonance (NMR) Spectroscopy and Mass Spectrometry (MS) coupled to gas-chromatography (GC) or liquid-chromatography (LC), it is increasingly popular to collect dynamic or time-resolved (or longitudinal) metabolomics data from biological systems. This is more so since such data holds the promise to be able to reveal underlying biological processes and mechanisms. Examples are from the field of metabolism and health, where challenge tests are used to probe the health status of individuals [1]; from food science where the metabolic fate of certain food compounds are studied [2]; in the study of diseases where biomarkers for diseases and early transitions to disease states are captured [3], and so on.

The main characteristics of the mentioned dynamic metabolomics studies are the limited number of time points at which measurements are taken from a limited number of subjects, and the superposition of different sources of variations. In terms of different sources of variation, first, there is induced variation which can be caused by different treatments, e.g., the Qingkailing injection group considered in [4], or caused by a disease whereby one enzyme has a much lower than usual activity, e.g., the human mutants described in [5]. Secondly, there is individual (also called biological) variation which is usually quite large [6]. Finally, there is (unavoidable) measurement error (also called technical error) which depends on the instrument and can be considerable [7]. All of these make the analysis of such dynamic metabolomics data challenging.

Given these challenges, dimension reduction methods are promising approaches since they are ideal for noise reduction (e.g., dealing with measurement error) and for capturing primary underlying sources of variation (see Smilde et al. [8] for a review on different methods to analyze dynamic metabolomics data). Dimension reduction techniques use the fact that there is an underlying low dimensionality in the data, and prototypical examples of such methods for so-called two-way data, such as Principal Component Analysis (PCA) and Orthogonal Partial Least Squares (OPLS), have shown their power [9], with extensions to dynamic probabilistic PCA for longitudinal metabolomics data analysis [10]. When data has more than two modes such as *subjects*, *metabolites* and *time*, a multiway array (also referred to as a higher-order tensor) can be constructed rather than treating the data as a two-way array, and dimension reduction methods for multiway arrays, known as tensor factorizations [11–14] can be used to analyze such temporal data. Compared to two-way PCA-based methods previously used to analyze time-evolving metabolomics data, tensor factorizations have the promise to provide the underlying patterns in all modes simultaneously, e.g., patterns in *subjects*, *metabolites* and *time* modes. Tensor factorizations have been successfully used in analyzing time-evolving data in data mining for discussion tracking [15], temporal link prediction [16], analysis of data streams [17], neuroimaging data analysis [18–20], and the analysis of electronic health records [21]. However, the use of tensor methods in dynamic metabolomics analysis has so far been limited due to the lack of such longitudinal metabolomics data until recently, and due to the limited understanding of the performance of the methods in metabolomics. One exception is the use of the CANDECOMP/PARAFAC (CP) [22, 23] tensor model combined with ASCA (ANOVA-simultaneous component analysis) to study the effect of treatments in time on a toxicological insult in rats [24].

**Fig. 1:**
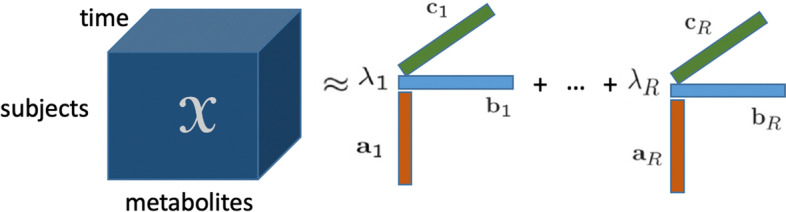
An *R*-component CP model of a three-way array with modes: *subjects*, *metabolites*, and *time*

In this paper, we explore the potential of tensor factorizations in analyzing dynamic metabolomics data and revealing the underlying mechanisms and their dynamics. To have the ground truth and study the limitations and advantages of such methods, we generate data through simulations of dynamic systems with increasing complexity, including a constructed linear open system, the yeast glycolysis model [25] and the human cholesterol model [5]. Both the glycolysis model and the cholesterol model are in silico models. These in silico models are realistic models of a biological system and allow for testing different scenarios of induced variation. To better mimic the real data, we introduce individual variation in these in silico models by randomly perturbing the kinetic parameters in the equations, and also introduce mutants, i.e., induced variation, by giving a decrease of specific parameters. We arrange the simulated data as a three-way array with modes: *subjects*, *metabolites*, and *time*, as shown in Fig. 1. The constructed multiway array is then analyzed using one of the most popular tensor models known as the CANDECOMP/PARAFAC model. We choose this model instead of other tensor models, e.g., the Tucker3 model [26], since the CP model is unique (up to permutation and scaling ambiguities) [13, 27]. Uniqueness leads to interpretable patterns which are important when analyzing dynamic metabolomics data. Moreover, we consider a restricted CP model, i.e., the Paralind (Parallel Profiles with Linear Dependences) model [28], since it can reveal the latent structure better than the CP model in the presence of linearly dependent factors.

## Methods

### Dynamic systems and data generation

The dynamics of metabolite concentrations can be modeled by differential equations of the form

1 $$\begin{aligned} \begin{aligned} \frac{d{\varvec{{x}}}}{dt}&=f({\varvec{{v}}}):={\varvec{{S}}}{\varvec{{v}}},\\ {\varvec{{x}}}(0)&={\varvec{{x}}}_0, \end{aligned} \end{aligned}$$

where the vector ${\varvec{{x}}}$ represents the metabolite concentrations, the derivative $\frac{d{\varvec{{x}}}}{dt}$ describes the change of metabolite concentrations over time, the vector ${\varvec{{v}}}$ describes the fluxes of reactions between the metabolites, and the matrix ${\varvec{{S}}}$ is the stoichiometric matrix describing the metabolic network. Each row in the matrix ${\varvec{{S}}}$ represents a metabolite, each column corresponds to a reaction, and each entry stands for the stoichiometric coefficient of a metabolite in a reaction for which a negative coefficient will be obtained with the metabolite consumed while a positive number will be given with the metabolite produced. The vector ${\varvec{{v}}}$ is usually a function of the concentrations of the metabolites with kinetic parameters.

#### Linear open system

If the fluxes are linear functions of concentrations: $f({\varvec{{v}}})={\varvec{{A}}}{\varvec{{x}}}+{\varvec{{b}}}$, then the differential equation can be rewritten as $\frac{d{\varvec{{x}}}}{dt} = {\varvec{{A}}}{\varvec{{x}}}+{\varvec{{b}}}$. We build a linear open system with 11 internal metabolites, where ${\varvec{{b}}}=10^3\times [0.1,0,0,0,0,0,0,0,0,0,0]^T$ and ${\varvec{{A}}}$ is a tridiagonal matrix of size $11\times 11$. The subdiagonal elements in matrix ${\varvec{{A}}}$ are set to be $10^3\times [0.2, 0.1, 0.5, 0.3, 2, 1, 3, 0.4, 1, 0.4]^T$ and the superdiagonal elements are set to $10^3 \times [0.3, 0.5, 2, 2, 0.3, 3, 0.5, 1, 0.2, 0.4]^T$. In addition, to satisfy the mass conservation law, the diagonal elements are chosen such that the summation of each column is zero except that ${\varvec{{A}}}(11,11)=-10^3$. The initial value is set to ${\varvec{{x}}}_0=[1, 1, 1, 1, 1, 1, 1, 1, 1, 1, 1]^T$. More details about the linear open system can be found in Additional file 1: Section 1. When we generate the data, we consider the simulation on [0, 0.2] min and pick the solution at time points $(6+5\times k)\times 0.002$ for $k=0,1,\dots ,19$. The pathway is shown in Additional file 1: Fig. S1.

#### Glycolysis model

The glycolysis model was proposed by Van Heerden et al. [25], and the non-linear term ${\varvec{{v}}}$ in Eq. (1) contains parameters describing the kinetic equations. This model is an open system, but much more complex than the linear open system due to the additional loops, e.g., the feed-forward control loop from metabolite FBP to enzyme PYK, the ADP-ATP cycle, and the NADH-NAD cycle shown in the pathway plot in Additional file 1: Fig. S3; more details about this model can be found in Additional file 1: Section 2. When we generate the data, we use the default initial values considered in [25]. We consider the simulation on [0, 0.2]min^1^, and pick the solution at time points $(6+5\times k)\times 0.002$ for $k=0,1,\dots ,19$.

#### Cholesterol model

The cholesterol model was proposed by van de Pas et al. [5], and the non-linear term ${\varvec{{v}}}$ in Eq. (1) for this model contains parameters in the kinetic equations. Similar to the glycolysis model, this model is also an open system but with more cycles among different cholesterols; see the pathway in Additional file 1: Fig. S8. The model was validated by data with ten known mutations, including, for example, the mutations that cause familial hypercholesterolemia (FH), fish eye disease, Smith–Lemli–Opitz syndrome (SLOS), and other diseases [5]. For each mutation, some particular enzymes have much lower activities than in the usual situation. In this paper, we consider these different types of mutants as different sources of induced variations. We generate the data by simulating the model using the same initial settings as in [5], i.e., all normal subjects start with the given initial metabolite conditions and the mutant subjects start with the steady state conditions of the normal subjects. The way we pick the time points is as follows: for the time points used in [5], i.e., (logspace(0,6,1000)-1)^2^, we start from the first time point and pick every 24th time point until we obtain 21 time points in total^3^.

### Multiway data analysis

#### CANDECOMP/PARAFAC (CP) model

The CP model, which stems from the polyadic form of a tensor [30], has become popular since it was introduced in 1970 [22, 23]. The CP factorization represents a tensor as a sum of rank-one tensors (see Fig. 1) and can be viewed as one generalization of the matrix Singular Value Decomposition (SVD). Given a third-order tensor $\varvec{\mathscr {X}}\in {\mathbb {R}}^{I\times J\times K}$, an *R*-component CP model of $\varvec{\mathscr {X}}$ is as follows:

$$\begin{aligned} \varvec{\mathscr {X}}\approx \varvec{\hat{\mathscr {{X}}}}=\llbracket {\varvec{{\lambda }}};{\varvec{{A}}}, {\varvec{{B}}},{\varvec{{C}}} \rrbracket :=\sum _{r=1}^R\lambda _r{\varvec{{a}}}_{r}\circ {\varvec{{b}}}_{r}\circ {\varvec{{c}}}_{r}, \end{aligned}$$

where the rank-one components consist of vectors ${\varvec{{a}}}_{r}, {\varvec{{b}}}_{r}$ and ${\varvec{{c}}}_{r}$ which are the columns of factor matrices ${\varvec{{A}}}\in {\mathbb {R}}^{I\times R}, {\varvec{{B}}}\in {\mathbb {R}}^{J\times R}$ and ${\varvec{{C}}}\in {\mathbb {R}}^{K\times R}$, respectively; $\lambda _r$ is a scalar, and $\circ$ denotes the vector outer product. In this definition, it is assumed that columns of ${\varvec{{A}}}, {\varvec{{B}}},{\varvec{{C}}}$ are normalized to norm one, and the weights are absorbed by the vector ${\varvec{{\lambda }}}$. Unlike most dimension reduction methods for two-way data sets, the CP model is unique up to permutation and scaling ambiguities under mild conditions, without imposing additional constraints [13, 27]. The uniqueness allows the CP model to give interpretable results, making it a much-preferred tool for interpretable data analysis. When interpreting the results, the factor loadings in the *subjects*, *metabolites* and *time* modes should be viewed together for each component.

The CP model can also be used to analyze data with missing entries [31, 32] by solving the following optimization problem:

$$\begin{aligned} \underset{A,B,C}{\text {min}} \left\Vert \, \varvec{\mathscr {W}}*(\varvec{\mathscr {X}}-\llbracket {\varvec{{\lambda }}};{\varvec{{A}}}, {\varvec{{B}}},{\varvec{{C}}} \rrbracket ) \, \right\Vert^2 , \end{aligned}$$

where the operator $*$ is the Hadamard product, $\left\Vert \, . \, \right\Vert$ denotes the Frobenius norm for higher-order tensors/matrices and the 2-norm for vectors, and entries of $\varvec{\mathscr {W}}\in {\mathbb {R}}^{I\times J\times K}$ are as follows:

2 $$\begin{aligned} {w}_{ijk} = {\left\{ \begin{array}{ll} 1 &\text{if $ {x}_{ijk}$ is known},\\ 0 &\text{if $ {x}_{ijk}$ is missing}. \end{array}\right. } \end{aligned}$$

#### Paralind model

For three-way data with patterns generated by underlying sources of variations with linearly dependent effects in at least one mode, the most appropriate CP model should show these dependences and such a solution is rank deficient. However, the standard CP model might fail to reveal the true latent structure due to the noise in the data [28]. Instead, a special case of CP model, namely the Paralind model [28] which was originally introduced as a restricted Tucker model [33], is more favourable. This model is partially unique, i.e., it has uniqueness only in the factors that have linearly independent factor vectors but non-uniqueness in the linearly dependent factors. It represents the implicit linear dependencies inherent in the data explicitly and thus recovers the latent structure more accurately. In addition, since fewer parameters are used in the Paralind model, it is less prone to overfitting. The Paralind model with linearly dependent factors in the first mode can be formulated as follows:

$$\begin{aligned} \varvec{\mathscr {X}}\approx \varvec{\hat{\mathscr {{X}}}}=\llbracket {\varvec{{\lambda }}};\tilde{{\varvec{{A}}}}, {\varvec{{B}}},{\varvec{{C}}} \rrbracket =\sum _{r=1}^R\lambda _r\tilde{\mathbf{a }}_{r}\circ {\varvec{{b}}}_{r}\circ {\varvec{{c}}}_{r}, \end{aligned}$$

where $\tilde{{\varvec{{A}}}}={\varvec{{A}}}{\varvec{{H}}}$ with ${\varvec{{A}}}\in {\mathbb {R}}^{I\times S}$ and ${\varvec{{H}}}\in {\mathbb {R}}^{S\times R}$, ${\varvec{{B}}}\in {\mathbb {R}}^{J\times R}$ and ${\varvec{{C}}}\in {\mathbb {R}}^{K\times R}$. The matrix ${\varvec{{H}}}$ is called the ‘dependency matrix’ which stores the linearly dependent relations. We denote this model by Paralind(*S*, *R*, *R*). Take a 3-component model with two components equal in the first mode as an example, the matrix ${\varvec{{H}}}$ in the Paralind(2,3,3) can be given as

$$\begin{aligned} {\varvec{{H}}}=\begin{bmatrix} 1 &{} 1 &{} 0\\ 0 &{} 0 &{} 1 \end{bmatrix}. \end{aligned}$$

## Numerical experiments

In this section, we first present the set-ups we used to generate the datasets and then demonstrate CP and Paralind models’ performance in terms of capturing the underlying mechanisms and dynamics.

### Experimental set-up and details

Before introducing the datasets, we first define the individual and induced variations.

- The *individual variation* refers to the random perturbations added to the constant kinetic parameters. The level of the individual variation (denoted by $\beta$) depends on the level of the perturbations. For the linear system, the individual variation is introduced by adding random perturbations to the superdiagonal and subdiagonal elements within a certain level, e.g., within 1%^4^ of the default values (i.e., $\beta =0.01$) and keeping the summations of each column to be zero except that ${\varvec{{A}}}(11,11)=-1\times 10^3$ is always enforced^5^ . For the glycolysis and cholesterol models, the individual variation is introduced by adding random perturbations within a certain level of the kinetic parameters, e.g., within 2% of the default values ($\beta =0.02$).
- The *induced variation* refers to the change given to a specific kinetic parameter and the level of the induced variation (denoted by $\alpha$) depends on the level of the change.

We consider the following two types of datasets.

- **Dataset with one source of induced variation.** This type of dataset contains 20 subjects:
  - (*Normal* subjects) The first 10 subjects are obtained by running simulations with only individual variation at level $\beta$;
  - (*Abnormal* subjects) The next 10 subjects are obtained by running simulations with individual variation at level $\beta$ and induced variation as giving a 50% decrease of the default value of ${\varvec{{A}}}(7,6)$ for the linear open systems (we denote these subjects by *abnormal_* ***A***(7,6) subjects); for the glycolysis model, as having a 50% decrease of the default values of VmaxPFK^6^ (these subjects are denoted by *abnormal_VmaxPFK* subjects); for the cholesterol model, as using mutant1 ($\alpha =0.62$) (these subjects are denoted by *abnormal_mutant1*^7^ subjects).
- **Dataset with two sources of induced variations.** This type of dataset contains 30 subjects and is generated for glycolysis and cholesterol models:
  - (*Normal* subjects) The first 10 subjects are generated in the same way as the *normal* subjects described above, with $\beta =0.02$.
  - (*Abnormal* subjects) The next 10 subjects are *abnormal_VmaxPFK* ($\alpha =0.50$) in the glycolysis model and *abnormal_mutant6* ($\alpha =0.35$) in the cholesterol model, all with $\beta =0.02$.
  - (*Abnormal* subjects) The last 10 subjects are *abnormal_VmaxPYK* ($\alpha =0.50$) in the glycolysis model and *abnormal_mutant10* ($\alpha =0.95$) in the cholesterol model, all with $\beta =0.02$.

Each dataset is then arranged as a third-order tensor with *subjects*, *metabolites* and *time* modes. Datasets generated by the linear open system and glycolysis model are of size # of subjects × 11 metabolites × 20 time points, and datasets generated by the cholesterol model are of size # of subjects × 8 metabolites × 21 time points.

#### Data preprocessing

Before the analysis, we center each third-order tensor across the *subjects* mode [34]. In addition, since concentrations of different metabolites are of different ranges, the tensor is scaled within the *metabolites* mode by the root mean squared value of each slice in the *metabolites* mode [34].

#### Model selection

When assessing different models and determining the number of components, we use several diagnostics, in particular, the model fit, core consistency diagnostic, cross-validation and Tucker’s congruence coefficient. The *model fit* (also often referred to as *explained variance*) is defined as:

$$\begin{aligned} \text {Fit}=100\times (1-\frac{\left\Vert \, \varvec{\mathscr {X}}-\varvec{\hat{\mathscr {{X}}}} \, \right\Vert ^2}{\left\Vert \, \varvec{\mathscr {X}} \, \right\Vert ^2}), \end{aligned}$$

where $\varvec{\mathscr {X}}$ and $\varvec{\hat{\mathscr {{X}}}}$ denote the original data and the data approximation by the model, respectively. A fit value of 100% means that $\varvec{\mathscr {X}}$ is fully explained by the model, while a fit value smaller than 100% implies that there is an unexplained part left in the residuals. An evident change in model fit for different models (e.g., models with different number of components) indicates a significant gain that should be considered when pursuing a better model.

The core consistency diagnostic has also been shown to be useful for determining the number of components in a CP model [35]. The *core consistency* of a CP model is defined by comparing the degree of superdiagonality of the core array^8^ of the CP model and the core array obtained by modeling the data with a Tucker3 model [26] using the CP factors. The core consistency value close to 100% indicates an appropriate model, and it is expected to drop if too many components are used.

Finally, we use missing data estimation performance through cross-validation for model selection. More precisely, we add some noise to the data, i.e.,

$$\begin{aligned} \varvec{\mathscr {X}}_\text {noise}=\varvec{\mathscr {X}}+\eta \varvec{\mathscr {N}}\frac{\Vert \varvec{\mathscr {X}}\Vert }{\Vert \varvec{\mathscr {N}}\Vert }, \end{aligned}$$

where $\varvec{\mathscr {N}}$ is a third-order tensor with entries randomly drawn from a standard normal distribution, and $\eta$ is the level of noise. We randomly set 20% of tensor entries to be missing, preprocess the data and use different models (i.e., CP and Paralind) to recover missing entries. We repeat this process 20 times to assess the performance of the methods using different sets of randomly missing entries. The performance of different models are then evaluated using the *tensor completion score* (TCS) defined as [31]

$$\begin{aligned} \text {TCS}=\frac{\Vert (1-\varvec{\mathscr {W}})*(\hat{\varvec{\mathscr {X}}}-\varvec{\mathscr {X}}_\text {noise})\Vert }{\Vert (1-\varvec{\mathscr {W}})*\varvec{\mathscr {X}}_\text {noise} \Vert }, \end{aligned}$$

where $\varvec{\mathscr {W}}$ is defined by Eq. (2). TCS can be viewed as an evaluation of the test error for a model and lower value indicates that the model behaves better in capturing the underlying patterns in the data.

CP models may suffer from a two-factor degeneracy (see [36] for more details on degeneracy). To assess whether the model has a two-factor degeneracy, we use the Tucker’s congruence coefficient (denoted by TC) [37]. The TC value for the *i*th and *j*th component is defined as:

$$\begin{aligned} \text {TC}_{ij} =\frac{{{\varvec{{a}}}_i}^T{\varvec{{a}}}_j}{\left\Vert \, {\varvec{{a}}}_i \, \right\Vert \left\Vert \, {\varvec{{a}}}_j \, \right\Vert }\frac{{{\varvec{{b}}}_i}^T{\varvec{{b}}}_j}{\left\Vert \, {\varvec{{b}}}_i \, \right\Vert \left\Vert \, {\varvec{{b}}}_j \, \right\Vert }\frac{{{\varvec{{c}}}_i}^T{\varvec{{c}}}_j}{\left\Vert \, {\varvec{{c}}}_i \, \right\Vert \left\Vert \, {\varvec{{c}}}_j \, \right\Vert }, \end{aligned}$$

which corresponds to the multiplication of cosine similarity ($C_{ij}=\frac{{{\varvec{{a}}}_i}^T{\varvec{{a}}}_j}{\left\Vert \, {\varvec{{a}}}_i \, \right\Vert \left\Vert \, {\varvec{{a}}}_j \, \right\Vert }$) of the two components in each mode. In this paper, we take the TC value as $\text {TC}=\text {TC}_{i_0j_0}$ where $|\text {TC}_{i_0j_0}|=\max \limits _{i,j}|\text {TC}_{ij}|$. A TC value close to − 1 indicates a degenerate model, which is not a valid model.

#### Implementation details

The CP models are fitted using cp-opt [38] and cp-wopt [31] (to data with missing entries) from the Tensor Toolbox version 3.1 [39] using Limited Memory BFGS with bounds (LBFGS-B)^9^ as the optimization algorithm. We impose non-negativity constraint in the *time* mode. The Paralind model^10^ is fitted using the algorithm introduced by Bro et al. [28]. In order to get unique models, we enforce the factor matrix in the *metabolites* mode to be orthogonal and in the *time* mode to be non-negative when fitting the Paralind model. Multiple random initializations are used to avoid local minima. For the computation of core consistency, we use the function corcond from the N-way toolbox [40]. All experiments are carried out in MATLAB (2020a release).

### Results and discussions

#### Linear open system

**Fig. 2:**
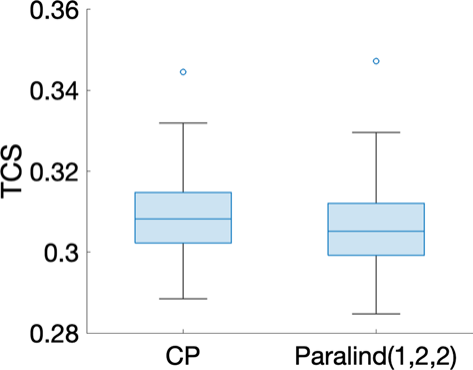
Cross-validation performance of the Paralind(1,2,2) model and the 2-component CP model for the data generated by the linear open system with one source of induced variation and individual variation at level $\beta =0.01$. The noise level is $\eta =0.3$, and 20 TCS values are used in the boxplots. The missing data patterns are the same for both CP and Paralind(1,2,2), and thus the TCS is paired. The difference in TCS is statistically significant based on the paired *t*-test

***Dataset with one source of induced variation*** We focus on the data with the induced variation as 50% decrease of the default value of ${\varvec{{A}}}(7,6)$, and the individual variation at level $\beta =0.01$. We first consider the analysis of the data using a CP model. From Table 1, we can see that the core consistency drops sharply from a 2-component model to a 3-component model. This implies that a 2-component model might be more suitable. However, rank deficiency is observed in the *subjects* mode for the 2-component CP model (the components in the *subjects* mode having a similarity score $C_{12}=1.00$). This indicates that the data indeed follows a Paralind(1,2,2) model, and the cross-validation performance shown in Fig. 2 implies that the Paralind(1,2,2) model is better than the 2-component CP model in recovering the left-out data.

**Table 1:** Explained variance (fit), core consistency (CC), Tucker’s congruence coefficient (TC), cosine similarity score of the first two components ($C_{12}$) in the *subjects* mode and number of components (*R*) for CP models used to analyze the data generated by the linear open system with one source of induced variation and individual variation at level $\beta =0.01$

*R*	Fit	CC	TC	$C_{12}$
1	88.15	100		
2	98.55	100	0.10	1.00
3	99.45	− 16	− 0.68

**Fig. 3:**
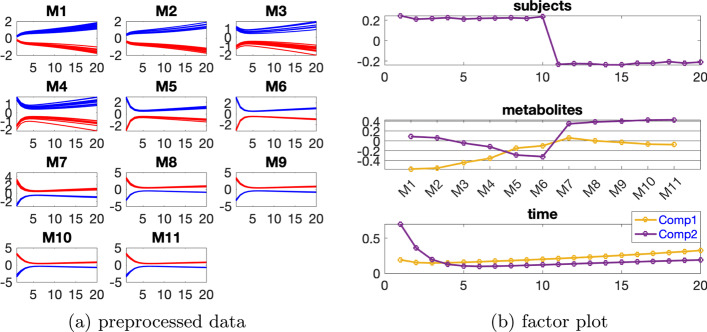
Data generated by the linear open system with one source of induced variation and the level of individual variation as $\beta =0.01$, and the factors captured using a Paralind(1,2,2) model. In Fig. 3a, legend: red (*normal*), blue (*abnormal_* ***A***(7,6))

The Paralind(1,2,2) model explains 98.38% of the data, which is slightly lower than the CP model due to the extra restriction. The subject mode of Paralind(1,2,2) model shows a clear separation between the two groups (Fig. 3b). From the first component in the *metabolites* mode (Fig. 3b), we observe that metabolites M1, M2, M3 and M4 have large absolute coefficients and in the *time* mode the first component captures the dynamics shown in these metabolites. The coefficients of the remaining metabolites for this component are close to zero. For the second component, oppositely, metabolites M7, M8, M9, M10, M11 and M5, M6 have large coefficients, and the dynamics shown in these metabolites are captured by the second component in the *time* mode. Besides, from both components in the *metabolites* mode (Fig. 3b), we observe a jump change between metabolites M6 and M7, which is consistent with the switch of the blue and red lines between these two metabolites shown in Fig. 3a. This change is due to the decrease of ${\varvec{{A}}}(7,6)$ in the *abnormal_* ***A***(7,6) subjects, and the successful capture of the change by the model results in the successful separation of the *normal* (the first 10 subjects) and *abnormal_* ***A***(7,6) (the last 10 subjects) groups in the *subjects* mode, as observed in Fig. 3b.

**Fig. 4:**
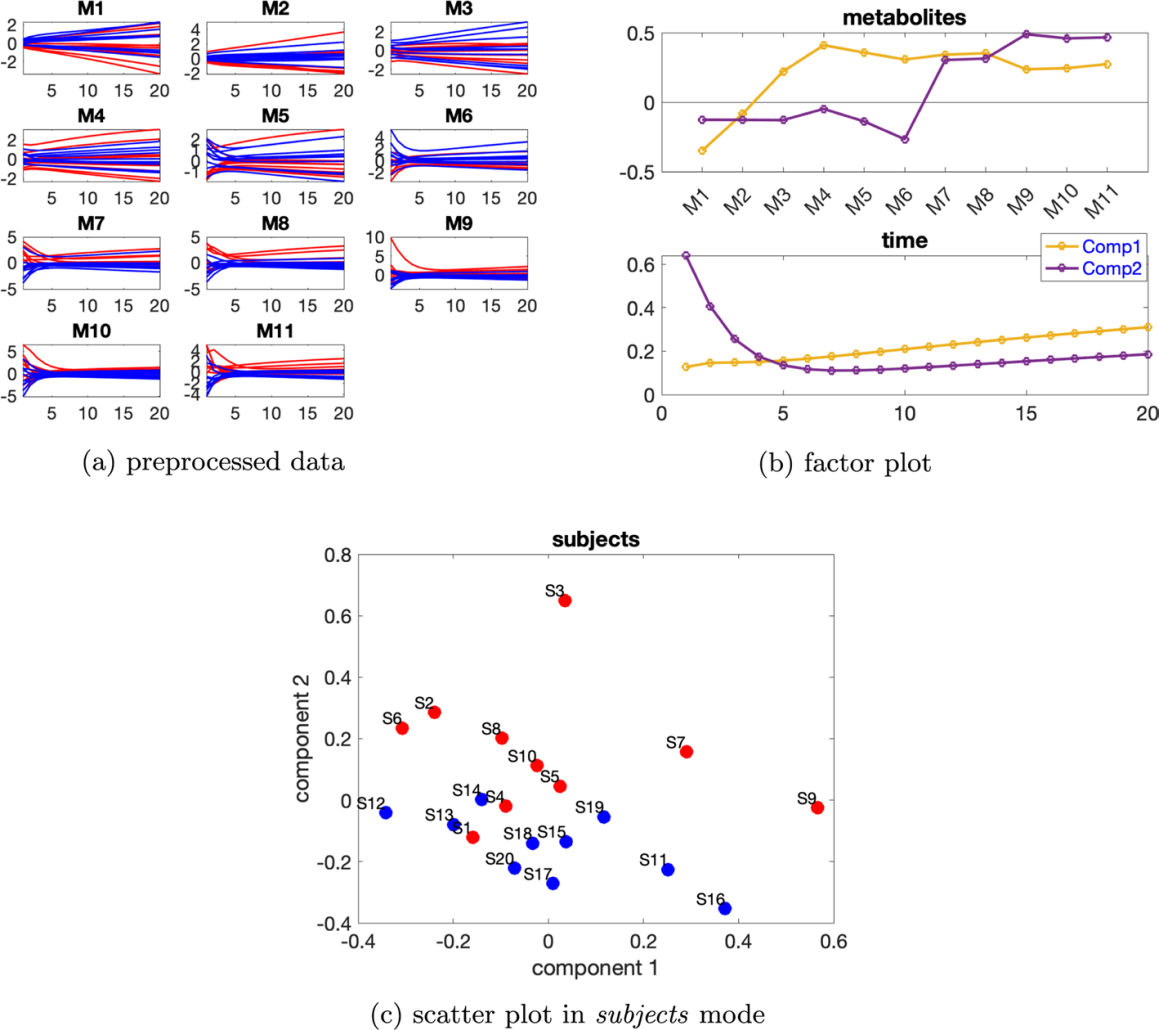
Data generated by the linear open system with one source of induced variation and the individual variation at level $\beta =0.3$, as well as the factors captured by a 2-component CP model. In Fig. 4a, c, legend: red (*normal*), blue (*abnormal_* ***A***(7,6))

When larger individual variation is considered, the rank deficiency disappears and CP models capture the underlying patterns better, see for example Fig. 4, where the level of the individual variation is $\beta =0.3$ and the similarity score of the two components in the *subjects* mode for the 2-component CP model is $C_{12}=-0.24$. The CP model explains 62.42% of the data. From the first component in the *metabolites* and *time* modes presented in Fig. 4b, we observe that all metabolites except for M2 have coefficients with large absolute values, and the component in the *time* mode captures the dynamic seen in all metabolites, to some extent. From the second component in the *metabolites* mode (4b), we observe that metabolites M6, M7, M8, M9, M10 and M11 have large coefficients, and in the *time* mode this component captures the fast decrease shown in these metabolites. The dynamics in M9, M10 and M11 are mainly captured by the second component, however the dynamics in metabolites M5, M6, M7 and M8 are a mixture of the two components in the *time* mode, as shown in Fig. 4a. Besides, we observe a jump change between metabolites M6 and M7 in the second component in the *metabolites* mode (4b), similar to the jump change shown in Fig. 3b. This is consistent with the switch of the blue and red lines in metabolites M6 and M7 shown in Fig. 4a and is due to the decrease of ${\varvec{{A}}}(7,6)$ in the *abnormal_* ***A***(7,6) subjects. Thus it is reasonable that the second component in the *subjects* mode can separate to some extent the *normal* and *abnormal_* ***A***(7,6) subjects, as shown in Fig. 4c.

Auxiliary experiments indicate that the behaviour of CP models relies on the kinetic coefficients. For some particular cases, degeneracy is observed for CP models, e.g., the setting with ${\varvec{{b}}}=10^3\times [0.5,0,0,0,0,0,0,0,0,0,0]^T$, and matrix ${\varvec{{A}}}$ a tridiagonal matrix for which the diagonal elements are set to be $10^3\times [-1, -2, -2, -2, -2, -2, -2, -2, -2, -2, -2]$ and the superdiagonal and subdiagonal elements are set to be $10^3\times [1, 1, 1, 1, 1, 1, 1, 1, 1, 1]$. For data generated by the linear open system with such a setting and a small individual variation, e.g, $\beta =0.01$, the CP model is degenerate. However, the Paralind model is useful in such cases as well and captures the underlying dynamics; see Additional file 1: Fig. S2.

#### Glycolysis model

***Dataset with one source of induced variation***

**Fig. 5:**
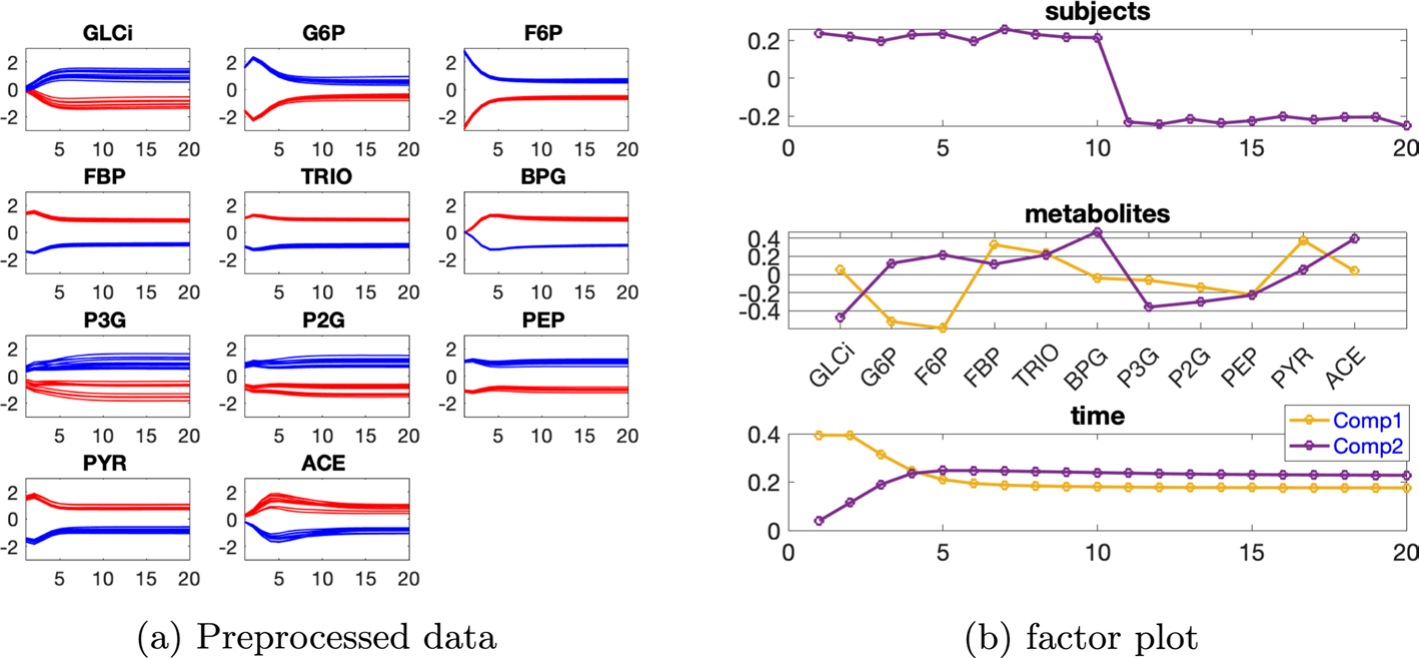
Data generated by the glycolysis model with one source of induced variation and individual variation at level $\beta =0.02$, and factors extracted from this dataset using a Paralind(1,2,2) model. In Fig. 5a, legend: red (*normal*), blue (*abnormal_VmaxPFK*)

We consider the data with the induced variation as 50% decrease of the default value of VmaxPFK, and the individual variation at level $\beta =0.02$. Temporal profiles of each metabolite are shown in Fig. 5a. Based on Table 2, we use a 2-component CP model, and as in the linear open system, we observe rank deficiency in the *subjects* mode. To account for rank deficiency, we instead use a Paralind(1,2,2) model to analyze this dataset. Cross-validation performance of CP versus Paralind also indicates that the Paralind(1,2,2) model, which explains 96.05% of the data, is a better choice for this dataset (see Additional file 1: Fig. S4). The two groups of subjects can be separated well and compared to the linear system, the factor plot in the *metabolites* mode shown in Fig. 5b is more complex due to the complexity of the network.

**Table 2:** Explained variance (fit), core consistency (CC), Tucker’s congruence coefficient (TC), cosine similarity score of the first two components ($C_{12}$) in the *subjects* mode and number of components (*R*) for CP models used to analyze the data generated by the glycolysis model with one source of induced variation and individual variation at level $\beta =0.02$

*R*	Fit	CC	TC	$C_{12}$
1	89.67	100		
2	96.31	100	0.06	0.99
3	98.17	− 5	− 0.74

The first component shows that there are two major jump changes, one between metabolites F6P and FBP and the other between metabolites PEP and PYR, which are consistent with the switch of the blue and red lines between these metabolites shown in Fig. 5a and are due to the decrease of VmaxPFK. The change between metabolites F6P and FBP corresponds directly to the decrease of VmaxPFK, similarly as the change shown in Fig. 3 for the linear open system. The change between metabolites PEP and PYR corresponds to the reduction of the activity of the enzyme VmaxPYK, which is due to the decrease of FBP caused by reducing VmaxPFK and the feed-forward control loop shown in the pathway plot (Additional file 1: Fig. S3). Metabolites G6P, F6P, FBP and PYR have large absolute coefficients on the first component and the dynamics from the third time points shown in these metabolites are well captured by the first component in the *time* mode, as demonstrated in Fig. 5b. The bumps shown in metabolites FBP and PYR can be captured by the linear combinations of the two components in the time mode. Models with an extra component will be helpful for capturing more variance, e.g., the bump in G6P, as illustrated in Additional file 1: Fig. S5. However, we prefer to use the Paralind (1,2,2) model since it captures most of the dynamic variations and is easier to interpret.

The second component in the *metabolites* mode indicates a jump change between metabolites BPG and P3G, which is consistent with the switch of the blue and red lines shown in Fig. 5a. This switch results from the increase of PEP, P2G and P3G caused by the drop of the reaction rate of VmaxPYK^11^, and the decrease of FBP, TRIO and BPG due to the reduction of VmaxPFK for the *abnormal_VmaxPFK* subjects. Metabolites GLCi, BPG, and ACE have large absolute scores on the second component and the dynamics of these metabolites are well captured by the second component in the *time* mode, as shown in Fig. 5b. The dynamics shown in metabolites TRIO, P3G, P2G and PEP are a mixture of both components in the *time* mode.

**Fig. 6:**
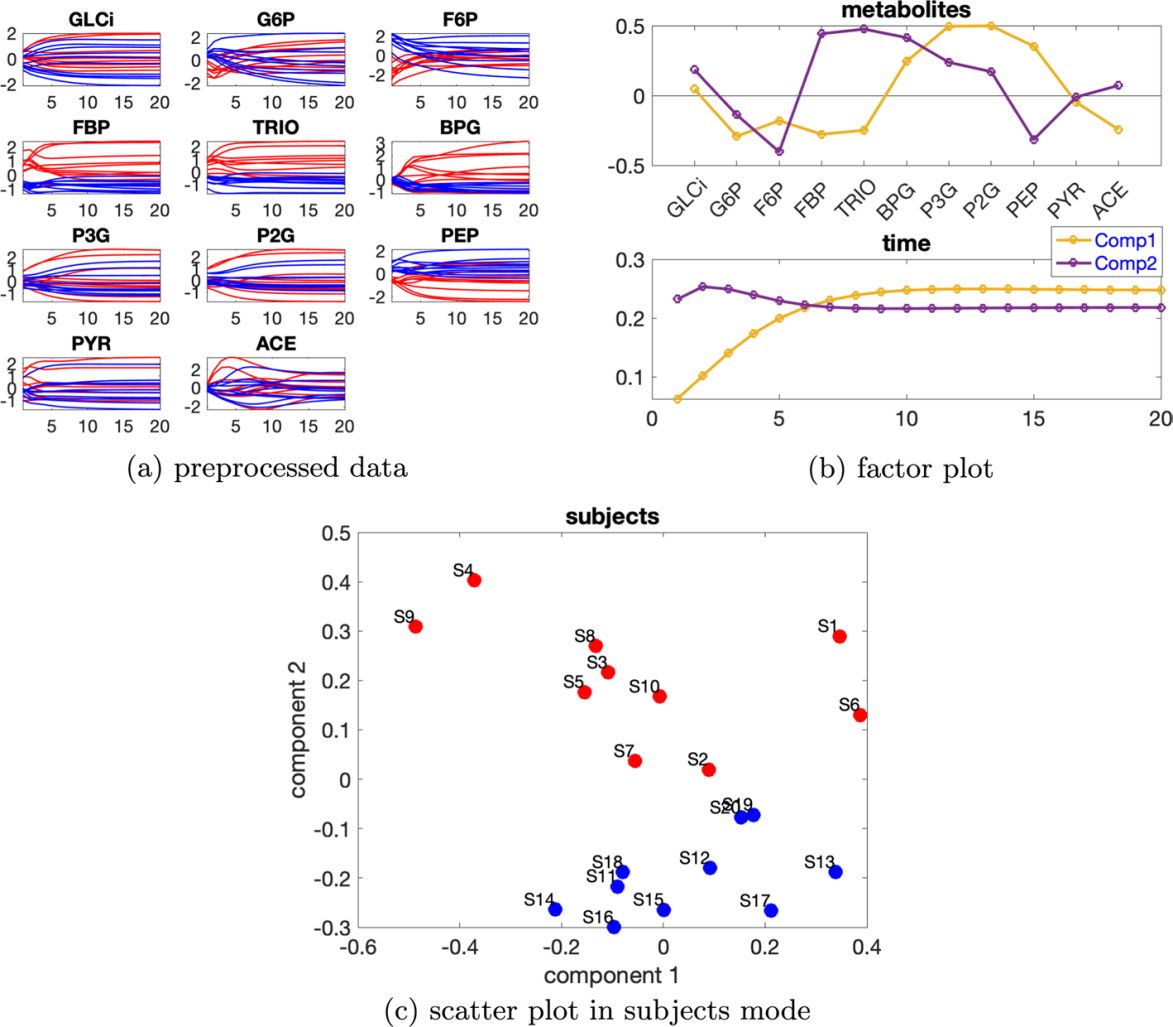
Data generated by the glycolysis model with one source of induced variation and the level of the individual variation as $\beta =0.36$, and the factors captured by a 2-component CP model. In Fig. 6a, c, legend: red (*normal*), blue (*abnormal_VmaxPFK*)

When a higher level of individual variation is considered, the linear dependence in the *subjects* mode gets weaker, see for example Additional file 1: Table S1, where the level of individual variation is $\beta =0.36$ and the cosine similarity score of the two components in the *subjects* mode for a 2-component CP model is $C_{12}=-0.28$. Thus CP models instead of Paralind models are preferable. From Additional file 1: Table S1, core consistency values indicate using a 2- or 3-component model. We choose the 2-component CP model since the additional factor in the 3-component model does not provide useful information. The 2-component CP model explains 54.12% of the data. From the first component in the *metabolites* and *time* mode (see Fig. 6b), we observe that metabolites P3G, P2G and PEP have large coefficients and the dynamics of those metabolites, as shown in Fig. 6a, are captured. From the second component in the *metabolites* mode (see Fig. 6b), we observe that metabolites F6P, FBP, TRIO and BPG have large absolute coefficients and in most of these metabolites the blue and red lines are separable. This is consistent with the separation observed in the *subjects* mode by the second component between the *normal* and *abnormal_VmaxPFK* subjects, as illustrated in Fig. 6c. The second component in the *time* mode captures the dynamics shown by some of the subjects in these metabolites (Fig. 6a).

For data with even larger individual variation, the CP models might not be able to separate the groups. The failure results from (i) the individual variation dominating the variance, see for example Additional file 1: Fig. S6, where the level of the individual variation is equal to the induced variation ($\beta =\alpha =0.50$), (ii) the limited number of subjects with the possibility of having one or two subjects showing idiosyncratic behavior (see the profiles for BPG in Additional file 1: Fig. S6a) and thus extracting commonality becomes challenging. Indeed, when the number of subjects is larger (see Additional file 1: Fig. S7), the 3-component CP model which explains 66.56% of the data can capture the main change (decrease of VmaxPFK) in the data and separate the *normal* and *abnormal_VmaxPFK* subjects successfully even for $\beta =\alpha =0.50$. Idiosyncratic behavior has become more common, thereby facilitating the modeling.

***Dataset with two sources of induced variations*** We consider the data generated with the individual variation at level $\beta =0.02$ and two sources of induced variation as 50% decrease of the default value for VmaxPFK and 50% decrease of the default value for VmaxPYK. Table 3 indicates using a 2-component CP model which explains 88.68% of the data.

**Table 3:** Explained variance (fit), core consistency (CC), Tucker’s congruence coefficient (TC), cosine similarity score of the first two components ($C_{12}$) in the *subjects* mode and number of components (*R*) for CP models used to analyze the data generated by the glycolysis model with two sources of induced variation as 50% decrease of VmaxPFK and 50% decrease of VmaxPYK as well as individual variation at level $\beta =0.02$

*R*	Fit	CC	TC	$C_{12}$
1	59.83	100		
2	88.68	100	− 0.00	0.09
3	96.04	1	− 1.00

**Fig. 7:**
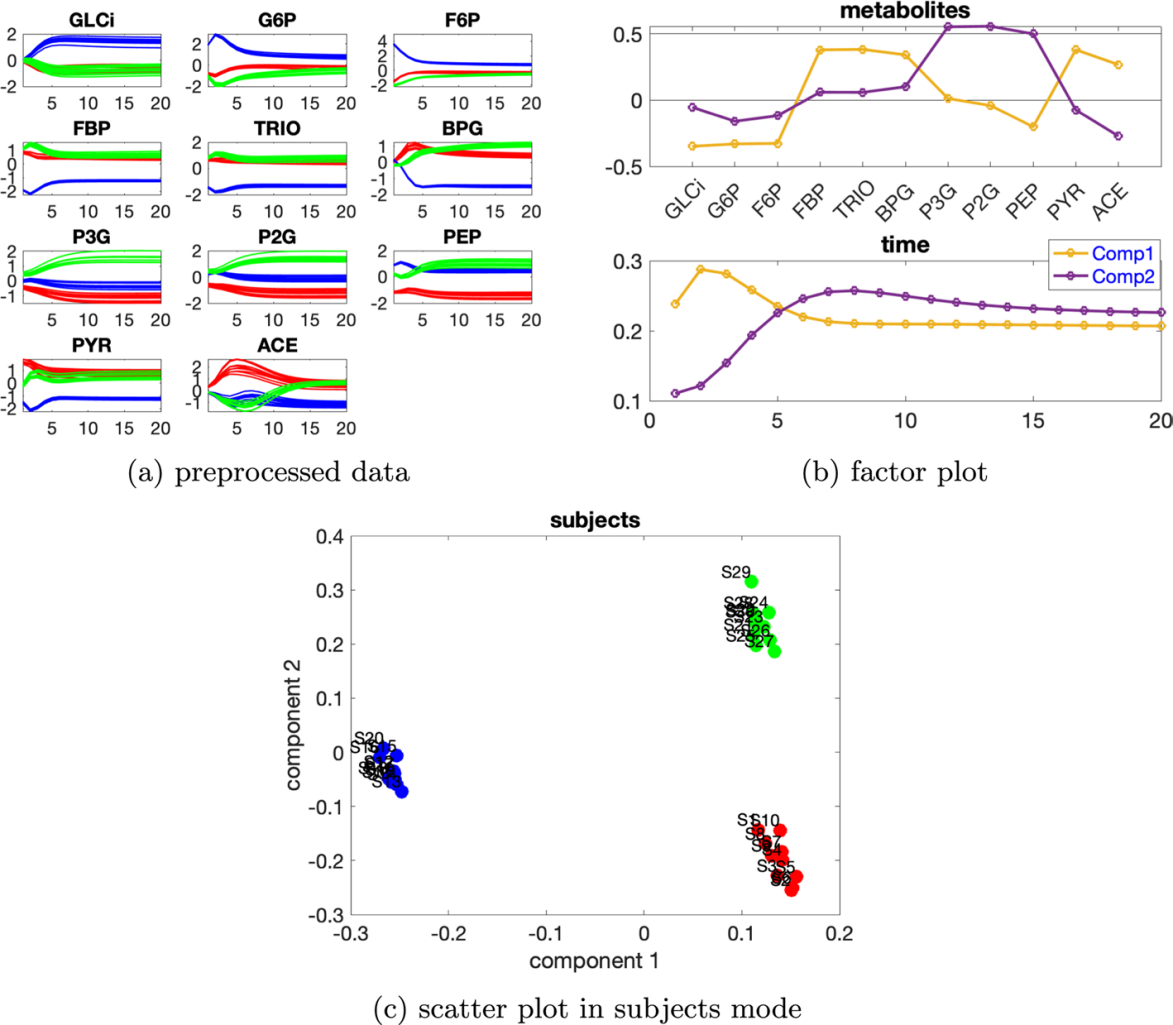
Data generated by the glycolysis model with the two sources of induced variations as 50% decrease of VmaxPFK and 50% decrease of VmaxPYK, and the individual variation at level $\beta =0.02$, as well as factors extracted from the data using a 2-component CP model. In Fig. 7a, c, legend: red (*normal*), blue (*abnormal_VmaxPFK*), green (*abnormal_VmaxPYK*)

From the first component in the *metabolites* and *time* mode (see Fig. 7b), we observe that metabolites GLCi, G6P, F6P, FBP, TRIO, BPG, PYR and ACE have large absolute coefficients and the dynamics in most of these metabolites, shown in Fig. 7a, are captured. Besides, the blue lines are separable from the other lines in these metabolites as shown in Fig. 7a. This is consistent with the observation that the first component in the *subjects* mode (Fig. 7c) separates the *abnormal_VmaxPFK* subjects from the others. Moreover, we observe a jump change between metabolites F6P and FBP and also between PEP and PYR, which is in accordance with the switch of the blue lines with the other lines in Fig. 7a due to the decrease of VmaxPFK and the feed-forward control loop. These observations are similar to what have been noticed in Fig. 5a, b for the glycolysis model with one source of induced variation. From the second component in the *metabolites* and *subjects* mode (see Fig. 7b, c), we see that metabolites P3G, P2G and PEP have large scores, and the three types of subjects can be separated from each other. This makes sense since different colors of lines are separable in metabolites P3G and P2G, as shown in Fig. 7a. Moreover, we observe a jump change between metabolites PEP and PYR on this component in the *metabolites* mode. This is compliant with the switch of the green lines with the other lines shown in Fig. 7a and it is due to the reduction of VmaxPYK. In the *time* mode, we observe that the dynamics shown in metabolites P3G and P2G are captured by the second component.

#### Cholesterol model

**Fig. 8:**
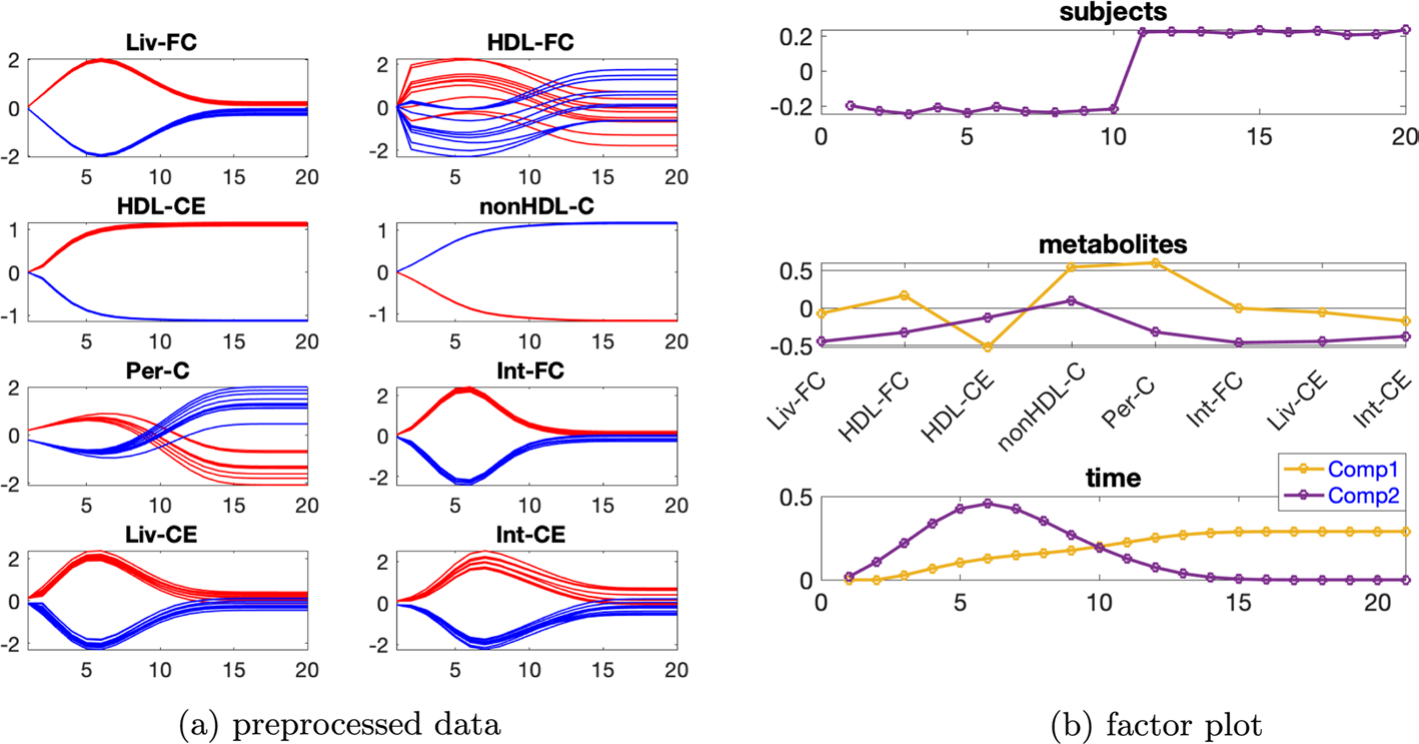
Data generated by the cholesterol model with one source of induced variation, and the individual variation at level $\beta =0.02$, as well as the factors extracted from this dataset using a Paralind(1,2,2) model. In Fig. 8a, legend: red (*normal*), blue (*abnormal_mutant1*)

***Dataset with one source of induced variation*** We consider the data with the induced variation as mutant1 and the individual variation at level $\beta =0.02$. Temporal profiles of the preprocessed data are shown in Fig. 8a. Additional file 1: Table S2 indicates using a 2- or 3-component model, and rank deficiency is observed in the *subjects* mode for CP models with both two and three components.

Thus we use the Paralind model, and from the interpretation, we prefer a 2-component model. Moreover, cross-validation performance (Additional file 1: Fig. S9) shows that the Paralind(1,2,2) model behaves better than the 2-component CP model. The Paralind(1,2,2) model explains 89.10% of the data. From the factor plot in the *subjects* mode (Fig. 8b), we observe a clear separation between the *normal* and *abnormal_mutant1* subjects. From the first component in the *metabolites* and *time* mode (see Fig. 8b), we observe that metabolites HDL-CE, nonHDL-C and Per-C have large coefficients while coefficients of the remaining metabolites are close to zero; the component in the *time* mode captures the dynamics shown in metabolite nonHDL-C and also a mixture of the dynamics in metabolites HDL-CE and Per-C, as illustrated in Fig. 8a. Moreover, we observe a clear jump change between metabolites HDL-CE and nonHDL-C which is consistent with the switch of the blue and red lines in Fig. 8a and is due to an elevation of metabolites nonHDL-C and a reduction of metabolites HDL-CE for *abnormal_mutant1* subjects caused by mutant1. From the second component in the *metabolites* and *time* mode (see Fig. 8b), we see that metabolites Liv-FC, Int-FC, Liv-CE and Int-CE have large coefficients and the component in *time* mode captures the common dynamics shown in these metabolites, as shown in Fig. 8a; we also observe a jump change between metabolites nonHDL-C and Per-C, which is consistent with the switch of the blue and red lines shown in Fig. 8a.

**Fig. 9:**
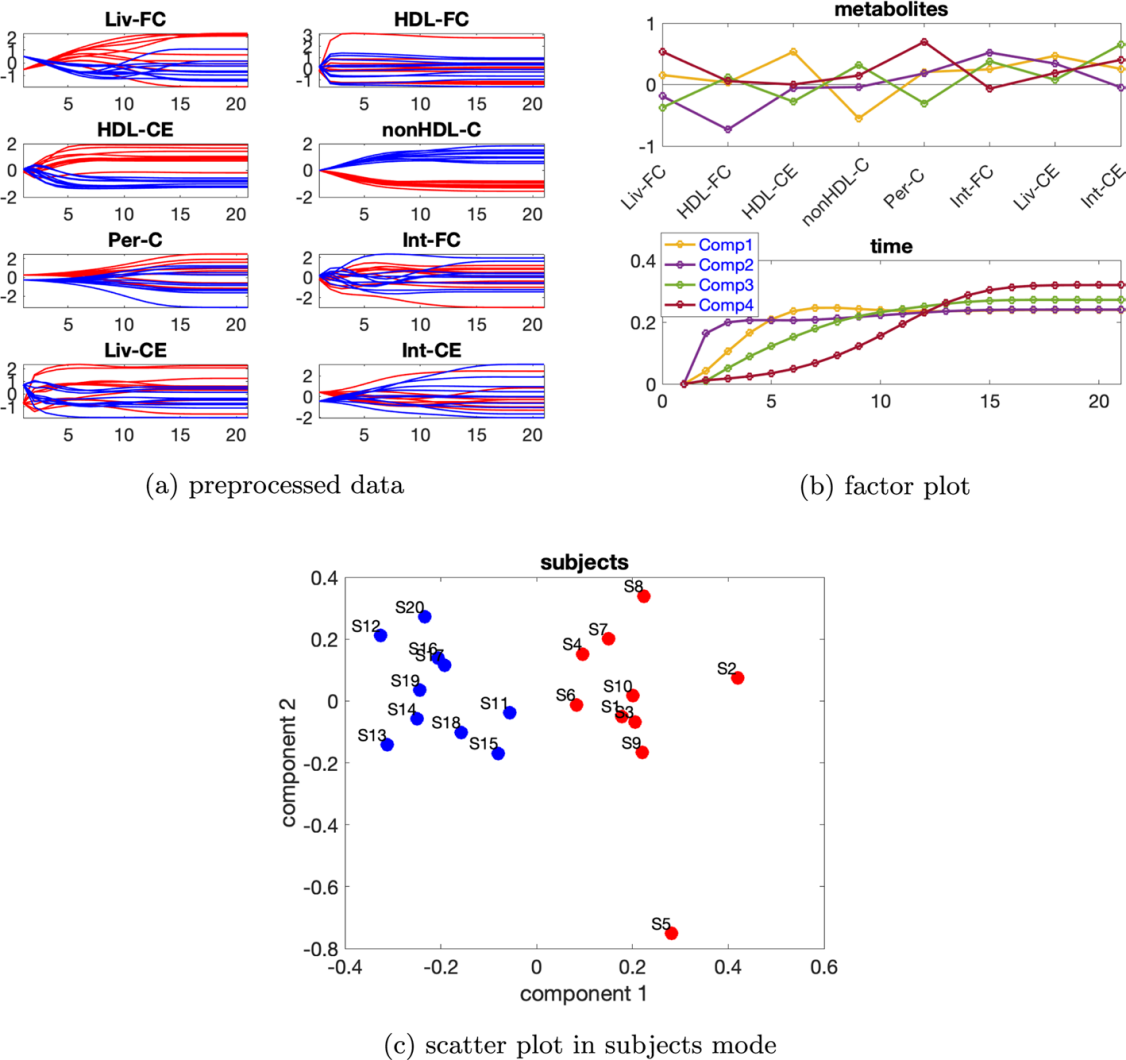
Data generated by the cholesterol model with one source of induced variation and the individual variation at level $\beta =0.65$, and the factors extracted from this dataset using a 4-component CP model. In Fig. 9a, c, legend: red (*normal*), blue (*abnormal_mutant1*)

This change is also due to mutant1 since the reaction rate from nonHDL-C to both Liv-FC and Per-C decreases which leads to a growth of nonHDL-C and a decrease of Per-C.

When high levels of individual variations are considered, rank deficiency in the *subjects* mode disappears, and CP models are preferable. We consider data with the individual variation at level $\beta =0.65$. Based on Additional file 1: Table S3, we use a 4-component CP model which explains 79.15% of the data. From the first component in the *metabolites* and *time* mode (see Fig. 9a), we observe that metabolites HDL-CE and nonHDL-C have the largest absolute coefficients and the dynamics in metabolite HDL-CE are captured, as illustrated in Fig. 9b. In addition, we see in Fig. 9c that the first component in the *subjects* mode separates the *normal* and *abnormal_mutant1* subjects. This is reasonable since the blue and red lines are separable in metabolites HDL-CE and nonHDL-C, as shown in Fig. 9a. The second component in the *time* mode captures the dynamics shown in metabolite HDL-FC which has the most significant absolute score on the second component in the *metabolites* mode. The third component captures the dynamics shown in metabolite Int-CE which has the largest positive score on the third component in the *metabolites* mode, and the fourth component captures the dynamics shown in metabolite Per-C which has the largest positive score on the fourth component in the *metabolites* mode.

**Fig. 10:**
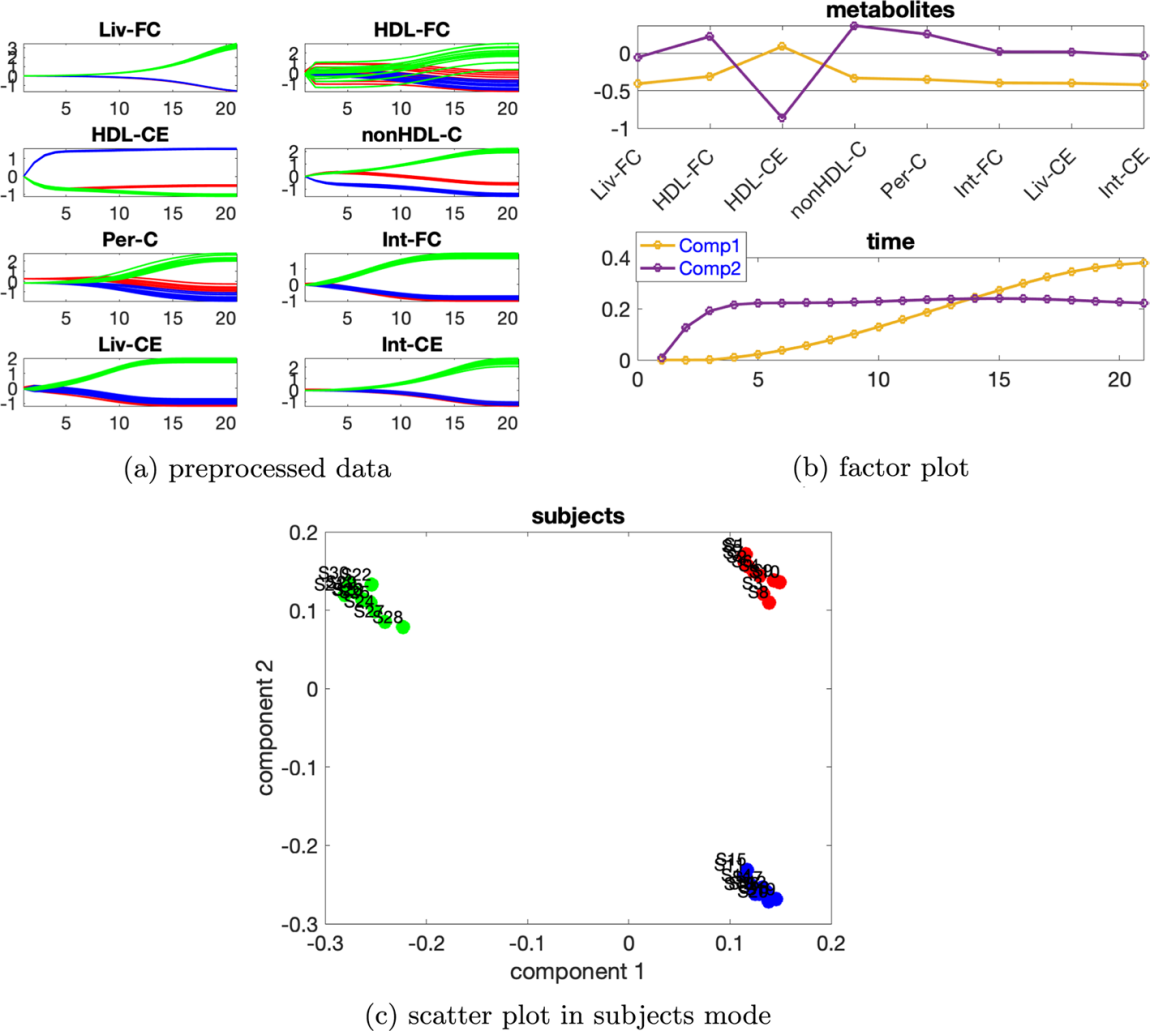
Data generated by the cholesterol model with the two sources of induced variations as mutant6 and mutant10 and the individual variation at level $\beta =0.02$, as well as the factors captured using a 2-component CP model. In Fig. 10a, c, legend: red (*normal*), blue (*abnormal_mutant6*), green (*abnormal_mutant10*)

***Dataset with two sources of induced variations*** We consider the data generated with the individual variation at level $\beta =0.02$ and two sources of induced variations as mutant6 and mutant10 in [5]. Additional file 1: Table S4 indicates using a 2-component model. The CP model with two components explains 91.89% of the data. From the *subjects* mode shown in Fig. 10c, we observe that the first component separates the *abnormal_mutant10* subjects from the remaining subjects while the second component separates the *normal* subjects from the *abnormal_mutant6* subjects. This makes sense since all the metabolites except for HDL-CE have a large coefficient on the first component in the *metabolites* mode, and we can see from Fig. 10a that for these metabolites, the blue lines and the red lines in the preprocessed data are quite close and they are clearly separated from the green lines. While metabolite HDL-CE has the largest absolute score on the second component and the blue lines are clearly separable from the other lines for metabolite HDL-CE as shown in Fig. 10a. Combining the plots in the *metabolites* and *time* mode (Fig. 10b), we observe that the model captures two main types of dynamics, i.e., one that increases fast to the steady state (the second component) shown in metabolite HDL-CE and one that increases slowly towards the steady state (the first component) shown in most of the remaining metabolites.

## Conclusion

In this paper, we have explored tensor factorizations for analyzing dynamic metabolomics data generated through simulations of dynamic systems. The basic idea for such methods, including the CP and Paralind model, is to extract the commonality between the subjects, i.e., the common dynamic behaviors. The dynamic behavior of metabolic systems as encountered in practice depends on (i) sizes of different sources of variation, and (ii) the structure of the system itself, i.e., the topology of the metabolic network as well as sizes of the kinetic constants. Using dynamic systems of increasing complexity, namely, a linear open system, a yeast glycolysis model and a human cholesterol model, we have studied the structure of the system as well as different sources of variation, and demonstrate how well CP and Paralind models capture the underlying dynamics in different settings. In all cases of enough commonality that we have studied, we can model the three-way data with relatively simple multiway models, i.e., the CP and Paralind models. These models manage to detect the interventions in the data, which is reflected by the successful capture of the changes in relations between metabolites, as shown by the jump changes in the factor plots of the metabolites. A detailed account of the relationship between the metabolic network (topology and connection strengths) and the factor loadings of the metabolites in CP or Paralind models is the subject of follow-up research. In most cases, we can also explain and understand the extracted patterns from the underlying in silico model. However, individual differences in dynamic behavior can be enormous in practice, e.g., in challenge tests [41]. This means that in a limited number of sampled individuals, there will be some with idiosyncratic behavior. We have demonstrated in our experiments that this idiosyncratic behavior is more of an undersampling problem.

The choice between a CP and a Paralind model depends on the data characteristics, and this, in turn, depends on the two aspects (i) and (ii) discussed in the above paragraph. In this paper, we present good diagnostics to select a proper model in practice. For data with small individual variation and sources of induced variations that have similar effects on the dynamic behavior, we use the Paralind model (due to linear dependence factors in the CP model); for data with large individual variation or data with various induced variations, we demonstrate that CP models work well.

For more complex cases such as dynamic systems with delays or with different dynamics due to significant differences in induced variation or with large idiosyncratic behavior, we may need more complex multiway models such as PARAFAC2 [42] or Restricted Tucker [33]. Also, for cases, where we are interested in time-evolving metabolites [10], PARAFAC2 is expected to reveal those by capturing evolving factor matrices in the *metabolites* mode. It may also be worth considering mixed effect three-way models accounting for the random variation among the individuals.

This simulation study is motivated by the analysis of a real dynamic metabolomics dataset. In real data, the underlying dynamic network is unknown and the data set size is larger, e.g., the number of metabolites and subjects is in the order of hundreds. CP models are still expected to reveal the main patterns of variations as well as the corresponding temporal profiles, as we plan to demonstrate with our findings on a real metabolomics challenge test dataset. The methods could also be scaled up to larger data sets [43, 44] (with thousands of or more variables in each mode) if such large-scale dynamic metabolomics data sets were to be available in the future.

While we focus on only the analysis of dynamic metabolomics data in this paper, future work includes joint analysis of multiple omics data sets [45] through extensions of tensor factorizations to coupled matrix and tensor factorizations [46].

## Supplementary Information

**Additional file 1:** **Figure S1.** Pathway of the linear open system. **Figure S2.** Time profiles for the data generated by the linear open system with a specific setting of parameters and the factor plot of the Paralind(1,2,2) model for this dataset. **Figure S3.** Pathway of the glycolysis model. **Figure S4.** Cross-validation performance of the 2-component CP model and the Paralind(1,2,2) model for data generated by the glycolysis model with one source of induced variation and the level of the individual variation as 0.02. **Figure S5.** Comparison of the true and predicted data by Paralind(1,2,2) and Paralind(1,3,3) for metabolite G6P. **Figure S6.** Time profiles for the data generated by the glycolysis model with one source of induced variation and the level of the individual variation as 0.5, as well as the factor plot of the 2-component CP model for this dataset. **Figure S7.** Time profiles for the data generated by the glycolysis model with 100 subjects and the level of the individual variation as 0.5, as well as the factor plot of the 3-component CP model for this dataset. **Figure S8.** Pathway of the cholesterol model. **Figure S9.** Cross-validation performance of the 2-component CP model and the Paralind(1,2,2) model for data generated by the cholesterol model with one source of induced variation and the level of the individual variation as 0.02. **Table S1.** Model selection information, including fit, CC, TC, *C*_12_ values, for CP models applied to the data generated by the glycolysis model with one source of induced variation and the level of the individual variation as 0.36. **Table S2.** Model selection information, including fit, CC, TC, *C*_12_ values, for CP models applied to the data generated by the cholesterol model with one source of induced variation and the level of the individual variation as 0.02. **Table S3.** Model selection information, including fit, CC, TC, *C*_12_ values, for CP models applied to the data generated by the cholesterol model with one source of induced variation and the level of the individual variation as 0.65. **Table S4.** Model selection information, including fit, CC, TC, *C*_12_ values, for CP models applied to the data generated by the cholesterol model with two sources of induced variations and the level of the individual variation as 0.02.

## Data Availability

Datasets used in the paper, and example scripts used for analyzing the datasets are available in the Github repository https://github.com/Lu-source/MultiwayAnalysis-DynamicMetabolomicsData.

## Footnotes

1 The reactions in the glycolysis model are very fast and the concentrations of metabolites reach the steady state quickly. Therefore, we focus on a short time interval where dynamic change shows up. However, it is feasible to acquire real metabolomics data at such a timescale; see in [29] where the sampling time can be 220 ms per sample when extracting the intracellular metabolites.

2 The time unit is day in the cholesterol model, and the time interval is set to be long enough in the experiment in [5] so that the system can reach its steady state.

3 In MATLAB notation: consider the vector tspan=logspace(0,6,1000)-1; the picked time points are tspan(1:24:500).

4 We choose small numbers to mimic systems with small biological variations, but this number can also be large as discussed later in the paper.

5 These restrictions are always used in the data generation for the linear open system to ensure the mass conservation law.

6 Here, enzymes other than VmaxPFK can also be considered. We choose VmaxPFK since we want to start with one enzyme positioned in the middle part of the pathway.

7 If the induced variation is defined by a decrease in other enzyme reaction rates, e.g., 50% decrease of VmaxPYK/mutant6/mutant10, then the *abnormal* subjects are denoted by *abnormal_VmaxPYK*/*abnormal_mutant6*/*abnormal_mutant10*, respectively.

8 The core array of a CP model is the core tensor obtained by expressing the CP model as a special case of a Tucker3 model. The core array of the CP model is a superdiagonal tensor with $\lambda$, i.e., weights of the rank-one components, on the superdiagonal, and all other entries as zero.

9 We use the implementation of LBFGS-B available on https://github.com/stephenbeckr/L-BFGS-B-C.

10 For Paralind model, we use the implementation on http://www.models.life.ku.dk/paralind.

11 The decrease of the reaction rate of VmaxPYK is due to the reduction of VmaxPFK which results in a decrease of FBP, and the effect of the feed-forward control loop.
