# Supplementary material for: Exploring dynamic metabolomics data with multiway data analysis: a simulation study
Source: BMC Bioinformatics. 2022 Jan 10;23:31. doi: 10.1186/s12859-021-04550-5 (PMC8750750; doi:10.1186/s12859-021-04550-5)
Supplement: Supplementary file 1 — Additional file 1: Figure S1. Pathway of the linear open system. Figure S2. Time profiles for the data generated by the linear open system with a specific setting of parameters and the factor plot of the Paralind(1,2,2) model for this dataset. Figure S3. Pathway of the glycolysis model. Figure S4. Cross-validation performance of the 2-component CP model and the Paralind(1,2,2) model for data generated by the glycolysis model with one source of induced variation and the level of the individual variation as 0.02. Figure S5. Comparison of the true and predicted data by Paralind(1,2,2) and Paralind(1,3,3) for metabolite G6P. Figure S6. Time profiles for the data generated by the glycolysis model with one source of induced variation and the level of the individual variation as 0.5, as well as the factor plot of the 2-component CP model for this dataset. Figure S7. Time profiles for the data generated by the glycolysis model with 100 subjects and the level of the individual variation as 0.5, as well as the factor plot of the 3-component CP model for this dataset. Figure S8. Pathway of the cholesterol model. Figure S9. Cross-validation performance of the 2-component CP model and the Paralind(1,2,2) model for data generated by the cholesterol model with one source of induced variation and the level of the individual variation as 0.02. Table S1. Model selection information, including fit, CC, TC, C12 values, for CP models applied to the data generated by the glycolysis model with one source of induced variation and the level of the individual variation as 0.36. Table S2. Model selection information, including fit, CC, TC, C12 values, for CP models applied to the data generated by the cholesterol model with one source of induced variation and the level of the individual variation as 0.02. Table S3. Model selection information, including fit, CC, TC, C12 values, for CP models applied to the data generated by the cholesterol model with one source of induced variation and the le [file 12859_2021_4550_MOESM1_ESM.pdf]

Supplementary material

1 Linear open system

1.1 Pathway

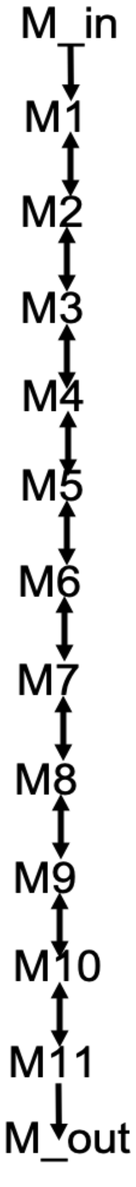

Figure 1: Pathway of the linear open system.

1.2 Details of the Linear open system

The linear open system, considered in the main text, has the form $\frac{d\mathbf{x}}{dt} = \mathbf{A}\mathbf{x} + \mathbf{b}$, where

$$
\mathbf{A} = 10^3 \times \begin{bmatrix}
-0.2 & 0.3 & 0 & 0 & 0 & 0 & 0 & 0 & 0 & 0 & 0 \\
0.2 & -0.4 & 0.5 & 0 & 0 & 0 & 0 & 0 & 0 & 0 & 0 \\
0 & 0.1 & -1 & 2 & 0 & 0 & 0 & 0 & 0 & 0 & 0 \\
0 & 0 & 0.5 & -2.3 & 2 & 0 & 0 & 0 & 0 & 0 & 0 \\
0 & 0 & 0 & 0.3 & -4 & 0.3 & 0 & 0 & 0 & 0 & 0 \\
0 & 0 & 0 & 0 & 2 & -1.3 & 3 & 0 & 0 & 0 & 0 \\
0 & 0 & 0 & 0 & 0 & 1 & -6 & 0.5 & 0 & 0 & 0 \\
0 & 0 & 0 & 0 & 0 & 0 & 3 & -0.9 & 1 & 0 & 0 \\
0 & 0 & 0 & 0 & 0 & 0 & 0 & 0.4 & -2 & 0.2 & 0 \\
0 & 0 & 0 & 0 & 0 & 0 & 0 & 0 & 1 & -0.6 & 0.4 \\
0 & 0 & 0 & 0 & 0 & 0 & 0 & 0 & 0 & 0.4 & -1
\end{bmatrix} \tag{1}
$$

and $\mathbf{b} = 10^3 \times [0.1, 0, 0, 0, 0, 0, 0, 0, 0, 0, 0]^T$. Regarding the pathway shown above in Fig 1, the considered linear open system can be rewritten as $\frac{d\mathbf{x}}{dt} = \mathbf{S}\mathbf{v}$, where the stoichiometric matrix $\mathbf{S}$ is a 11×22 matrix with the form

$$
\mathbf{S} = \begin{bmatrix}
1 & -1 & 1 & 0 & 0 & 0 & 0 & 0 & 0 & 0 & 0 & 0 & 0 & 0 & 0 & 0 & 0 & 0 & 0 & 0 & 0 & 0 \\
0 & 1 & -1 & -1 & 1 & 0 & 0 & 0 & 0 & 0 & 0 & 0 & 0 & 0 & 0 & 0 & 0 & 0 & 0 & 0 & 0 & 0 \\
0 & 0 & 0 & 1 & -1 & -1 & 1 & 0 & 0 & 0 & 0 & 0 & 0 & 0 & 0 & 0 & 0 & 0 & 0 & 0 & 0 & 0 \\
0 & 0 & 0 & 0 & 0 & 1 & -1 & -1 & 1 & 0 & 0 & 0 & 0 & 0 & 0 & 0 & 0 & 0 & 0 & 0 & 0 & 0 \\
0 & 0 & 0 & 0 & 0 & 0 & 0 & 1 & -1 & -1 & 1 & 0 & 0 & 0 & 0 & 0 & 0 & 0 & 0 & 0 & 0 & 0 \\
0 & 0 & 0 & 0 & 0 & 0 & 0 & 0 & 0 & 1 & -1 & -1 & 1 & 0 & 0 & 0 & 0 & 0 & 0 & 0 & 0 & 0 \\
0 & 0 & 0 & 0 & 0 & 0 & 0 & 0 & 0 & 0 & 0 & 1 & -1 & -1 & 1 & 0 & 0 & 0 & 0 & 0 & 0 & 0 \\
0 & 0 & 0 & 0 & 0 & 0 & 0 & 0 & 0 & 0 & 0 & 0 & 0 & 1 & -1 & -1 & 1 & 0 & 0 & 0 & 0 & 0 \\
0 & 0 & 0 & 0 & 0 & 0 & 0 & 0 & 0 & 0 & 0 & 0 & 0 & 0 & 0 & 1 & -1 & -1 & 1 & 0 & 0 & 0 \\
0 & 0 & 0 & 0 & 0 & 0 & 0 & 0 & 0 & 0 & 0 & 0 & 0 & 0 & 0 & 0 & 0 & 1 & -1 & -1 & 1 & 0 \\
0 & 0 & 0 & 0 & 0 & 0 & 0 & 0 & 0 & 0 & 0 & 0 & 0 & 0 & 0 & 0 & 0 & 0 & 0 & 1 & -1 & -1
\end{bmatrix},
$$

and the vector $\mathbf{v}$ satisfies

$$
\mathbf{v} = [v_{\text{in}}, v_{1,2}, v_{2,1}, v_{2,3}, v_{3,2}, \cdots, v_{i,i+1}, v_{i+1,i}, \cdots, v_{10,11}, v_{11,10}, v_{\text{out}}]^T,
$$

where

$$
\begin{aligned}
v_{\text{in}} &= \mathbf{b}(1), \\
&\vdots \\
v_{i,i+1} &= \mathbf{A}(i+1, i)x_i, \\
v_{i+1,i} &= \mathbf{A}(i, i+1)x_{i+1}, \\
&\vdots \\
v_{\text{out}} &= \mathbf{A}(11, 11)x_{11},
\end{aligned}
$$

for $i = 1, \cdots, 10$.

There are no particular rules for constructing the linear open system except that

- the first element of $\mathbf{b}$ should be a positive number which corresponds to the inflow of the system,
- the summation of the last column should be a negative number, i.e., $\mathbf{A}(11, 11)$ should be smaller than $-\mathbf{A}(10, 11)$, and the number $\mathbf{A}(10, 11) - \mathbf{A}(11, 11)$ corresponds to the outflow of the system.

The setting we use is just one choice and other settings are also allowed if the two rules are satisfied. The behaviour of the tensor factorization methods might differ when different settings of kinetic coefficients are used.

1.3 Paralind(1,2,2) model for a specific setting

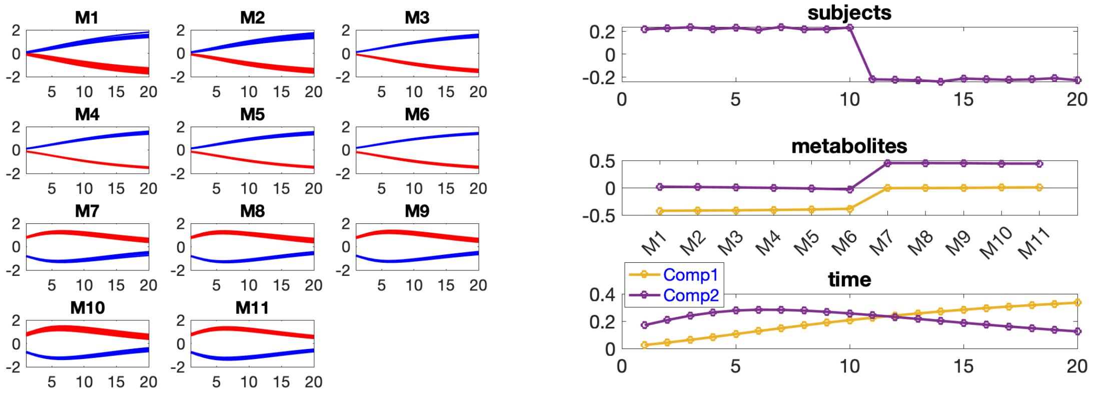

(a) preprocessed data

(b) factor plot

Figure 2: Data generated by the linear open system with $\mathbf{b} = 10^3 \times [0.5, 0, 0, 0, 0, 0, 0, 0, 0, 0, 0]^T$ and matrix $\mathbf{A}$ a tridiagonal matrix for which the diagonal elements are set to be $10^3 \times [-1, -2, -2, -2, -2, -2, -2, -2, -2, -2, -2]$ and the superdiagonal and subdiagonal elements are set to be $10^3 \times [1, 1, 1, 1, 1, 1, 1, 1, 1, 1]$, with one source of induced variation and the individual variation at level $\beta = 0.01$, as well as the factors captured using a Paralind(1,2,2) model. In Fig 2a, legend: red(*normal*), blue(*abnormal_ $\boldsymbol{A}$(7,6)*).

Interpretations of the results in Fig 2

From the factor plot in Fig 2b, we can see a clear separation of *normal* (the first 10 subjects) and *abnormal_ $\boldsymbol{A}$(7,6)* (the last 10 subjects) groups. Besides, we observe a jump change between metabolites `M6` and `M7`. This is consistent with the way the data is generated, i.e., 50% decrease of $\mathbf{A}(7, 6)$ that results in an increase of `M1` to `M6` and a decrease of the other metabolites if the *abnormal_ $\boldsymbol{A}$(7,6)* subjects are compared to the *normal* subjects. The factor plot in the *time* mode (Fig 2b) indicates that two types of dynamics are captured, i.e., the first component describing the behaviour of the first 6 metabolites and the second component describing the behaviour of the last 5 metabolites shown in Fig 2a. Please note that in the *metabolites* mode the first 6 metabolites have zero scores on the second component while the last 5 metabolites have zero scores on the first component.

2 Glycolysis model

2.1 Pathway

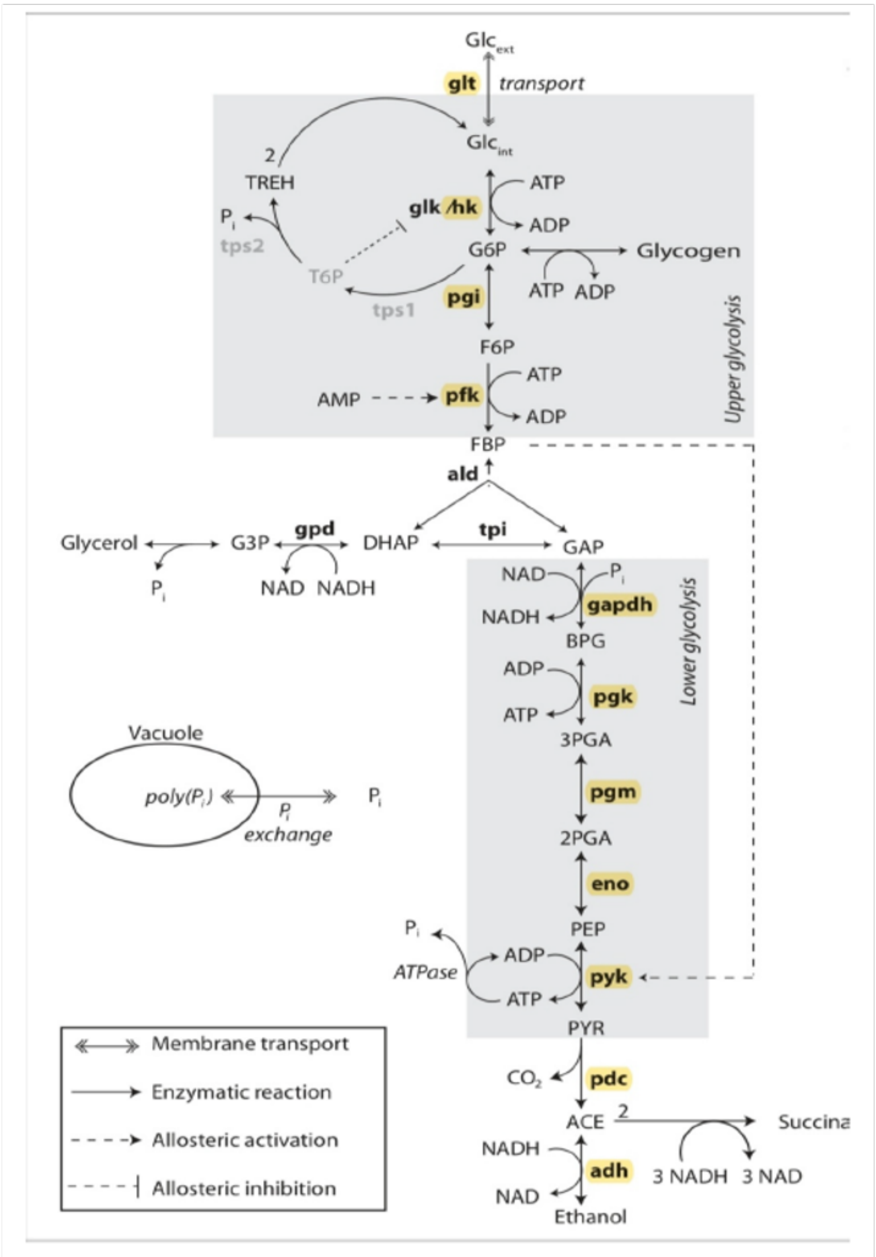

Figure 3: Pathway of the glycolysis model from [1] (see on https://science.sciencemag.org/content/343/6174/1245114), is reprinted with the permission from *AAAS*.

2.2 More details about the glycolysis model

Among the outputs of the glycolysis model [1], there are five metabolites that are not shown in the pathway plot in Fig 3, i.e., `TRIO`, `Prb`, `PHOS`, `P3G` and `P2G`. In fact, the metabolite `TRIO` in the model output corresponds to the metabolite `GAP` in the pathway since `GAP` can be calculated by `TRIO` (see equations 20 and 21 in [2] for detail); the metabolite `Prb` in the model output corresponds to the metabolite `ATP` in the pathway (see details on https://jjj.mib.ac.uk/models/vanheerden1/); the metabolite `PHOS` in the model output corresponds to the metabolite `Pi` in the pathway (both refer to the free phosphate, but they are named differently in the model and the pathway in [1]); the metabolites `P3G` and `P2G` in the model output correspond to the

metabolites 3PGA and 2PGA in the pathway.

Metabolites ATP, NADH, and PHOS take part in many reactions in many pathways, and it is not easy to interpret the behavior of these metabolites in practice. Therefore we do not include the concentrations of these metabolites in the data for further analysis.

2.3 Supplemental Tables and Figures

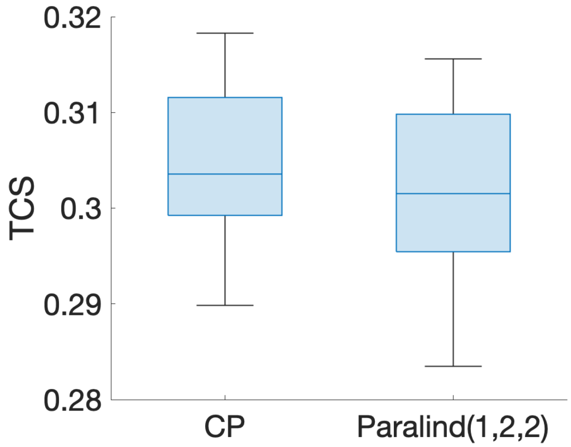

Figure 4: Cross-validation performance of 2-component CP model and the Paralind(1,2,2) model for data generated by the glycolysis model with one source of induced variation and individual variation at level $\beta = 0.02$. The noise level is $\eta = 0.3$, and 20 TCS values are used in the boxplots. The difference in TCS is statistically significant based on the paired t-test.

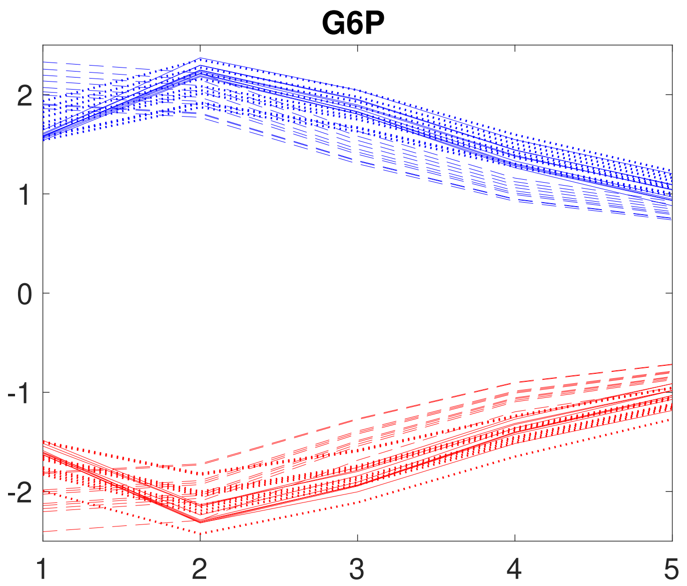

Figure 5: Comparison of the true data with the reconstructed data by Paralind(1,2,2) and Paralind(1,3,3) for G6P at the first 5 time points. **solid lines**: the true data; **dashed lines**: the reconstructed data by Paralind(1,2,2); **dashed dotted lines**: the reconstructed data by Paralind(1,3,3). legend: red(*normal*), blue(*abnormal_ VmaxPFK*). This figure corresponds to Figure 5 in the main text.

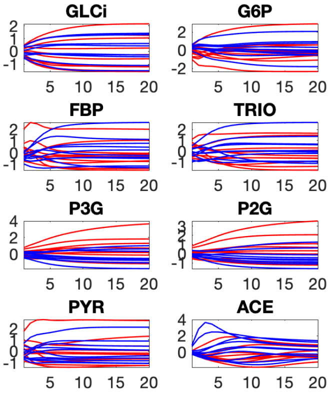

(a) preprocessed data

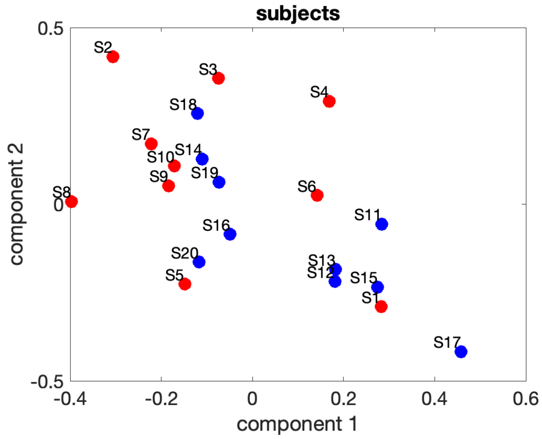

(b) scatter plot in subjects mode

Figure 6: Data generated by the glycolysis model with one source of induced variation and the level of the individual variation as $\beta = 0.50$, as well as the factors captured by the 2-component CP model. Legend: red($normal$), blue($abnormal\_ \ VmaxPFK$).

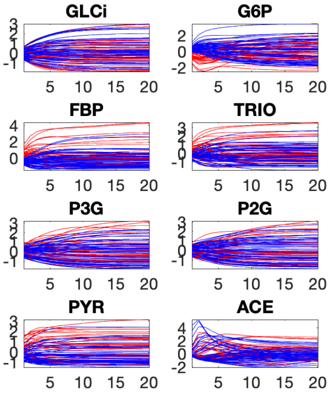

(a) preprocessed data

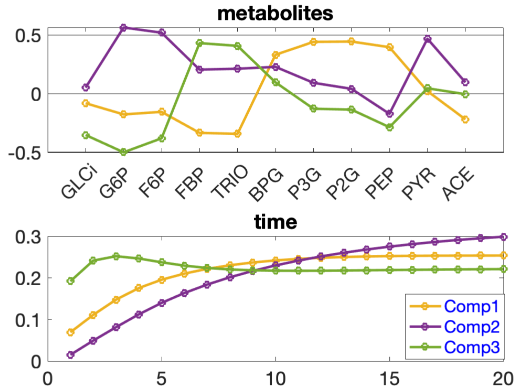

(b) factor plot

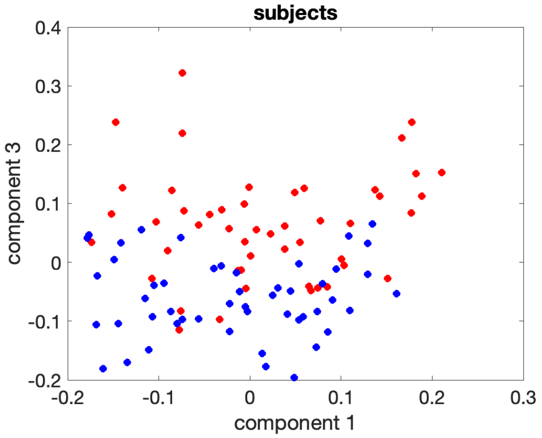

(c) scatter plot in subjects mode

Figure 7: Data generated by the glycolysis model with 100 subjects, one source of induced variation and the level of the individual variation as $\beta = 0.50$, as well as the factors captured by a 3-component CP model. In Fig 7a and 7c, legend: red($normal$), blue($abnormal\_ \ VmaxPFK$).

Table 1: Explained variance (fit), core consistency (CC), Tucker's congruence coefficient (TC), cosine similarity score of the first two components (C_{12}) in the *subjects* mode and number of CP components (R) for CP models applied to data generated by the glycolysis model with one source of induced variation and individual variation at level $\beta = 0.36$.

R	**fit**	**CC**	**TC**	C_{12}
1	35.11	100		
2	54.12	100	-0.01	-0.28
3	71.17	71	-0.53	
4	80.96	40	-0.39	
5	88.39	<0	-0.99	

3 Cholesterol model

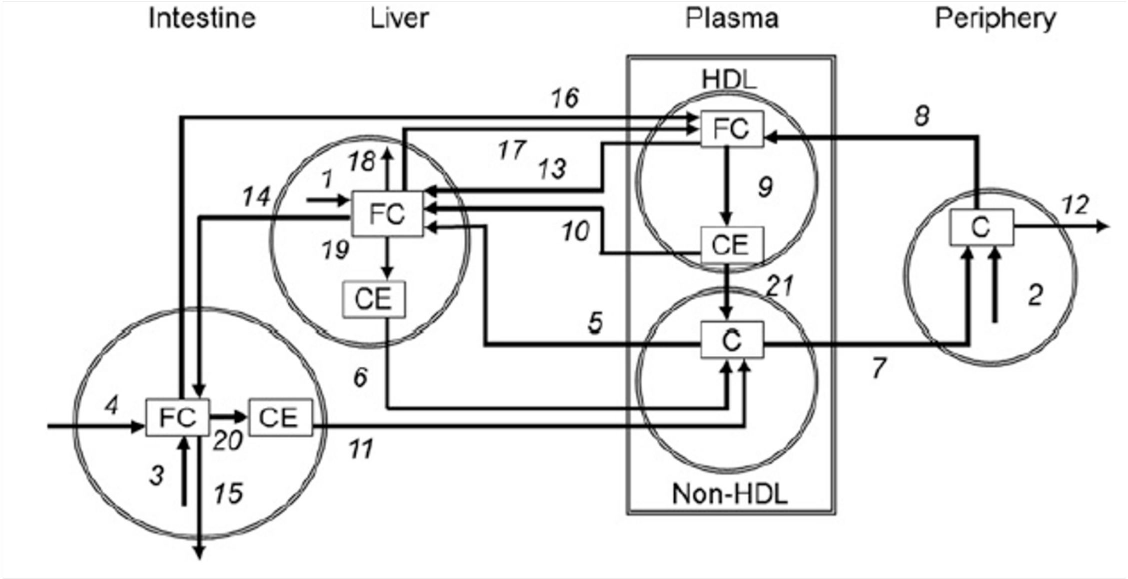

Figure 8: Pathway of the cholesterol model from [3]; it is reprinted with the permission license *Creative Commons CC-BY*. Mutations 1, 6 and 10 induce reduced reaction rates of reactions 5, 21 and 18, respectively.

3.1 Supplemental Tables and Figures

Table 2: Explained variance (fit), core consistency (CC), Tucker's congruence coefficient (TC), cosine similarity score of the first two components (C_{12}) in the *subjects* mode and number of CP components (R) for CP models applied to data generated by the cholesterol model with one source of induced variation and the individual variation at level $\beta = 0.02$.

R	**fit**	**CC**	**TC**	C_{12}
1	64.88	100		
2	90.68	100	-0.82	-1.00
3	97.40	89	-0.48	
4	98.99	54	-0.66	

Table 3: Explained variance (fit), core consistency (CC), Tucker's congruence coefficient (TC), cosine similarity score of the first two components (C_{12}) in the *subjects* mode and number of CP components (R) for CP models applied to data generated by the cholesterol model with one source of induced variation and the individual variation at level $\beta = 0.65$.

R	**fit**	**CC**	**TC**	C_{12}
1	33.76	100		
2	55.29	100	0.00	0.00
3	68.28	75	-0.02	
4	79.15	78	-0.05	
5	86.99	$<$0	-1.00	

Table 4: Explained variance (fit), core consistency (CC), Tucker's congruence coefficient (TC), cosine similarity score of the first two components (C_{12}) in the *subjects* mode and number of CP components (R) for CP models applied to data generated by the cholesterol model two sources of induced variations (the `mutant6` ($\alpha = 0.35$) and `mutant10` ($\alpha = 0.95$)), and the individual variation at level $\beta = 0.02$.

R	**fit**	**CC**	**TC**	C_{12}
1	82.30	100		
2	91.89	100	0.12	-0.44
3	96.07	11	-0.44	

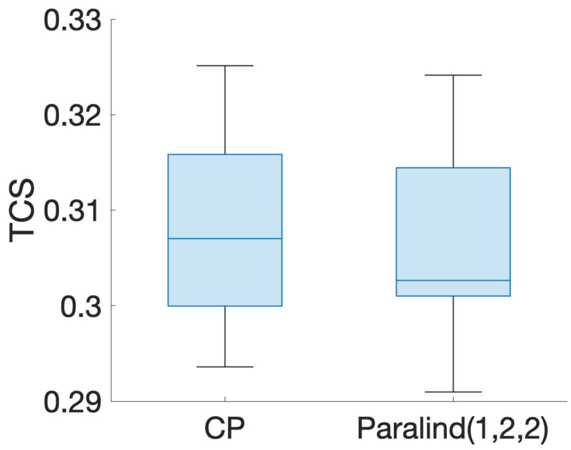

Figure 9: Cross-validation performance of the 2-component CP model and the Paralind(1,2,2) model for data generated by the cholesterol model with one source of induced variation and individual variation at level $\beta = 0.02$. The level of the noise is $\eta = 0.3$, and 20 TCS values are used in the boxplots. The difference in TCS is statistically significant based on the paired t-test.

References

[1] van Heerden, J.H., Wortel, M.T., Bruggeman, F.J., Heijnen, J.J., Bollen, Y.J., Planqué, R., Hulshof, J., O'Toole, T.G., Wahl, S.A., Teusink, B.: Lost in transition: start-up of glycolysis yields subpopulations of nongrowing cells. Science **343**(6174) (2014)

[2] Teusink, B., Passarge, J., Reijenga, C.A., Esgalhado, E., Van der Weijden, C.C., Schepper, M., Walsh, M.C., Bakker, B.M., Van Dam, K., Westerhoff, H.V., *et al.*: Can yeast glycolysis be understood in terms

of in vitro kinetics of the constituent enzymes? testing biochemistry. European Journal of Biochemistry **267**(17), 5313–5329 (2000)

[3] van de Pas, N.C., Woutersen, R.A., van Ommen, B., Rietjens, I.M., de Graaf, A.A.: A physiologically based in silico kinetic model predicting plasma cholesterol concentrations in humans. Journal of lipid research **53**(12), 2734–2746 (2012)
